# Cobalt(II) Complexes of 4′–Nitro–Fenamic Acid: Characterization and Biological Evaluation

**DOI:** 10.3390/molecules30122621

**Published:** 2025-06-17

**Authors:** Georgios Malis, Antigoni Roussa, Efstathia Aikaterini Papantopoulou, Stavros Kalogiannis, Antonios G. Hatzidimitriou, Konstantina C. Fylaktakidou, George Psomas

**Affiliations:** 1 Laboratory of Inorganic Chemistry, Department of Chemistry, Aristotle University of Thessaloniki, 54124 Thessaloniki, Greece; 2 Department of Nutritional Sciences and Dietetics, International Hellenic University, Sindos, 57400 Thessalonikis, Greece; 3 Laboratory of Organic Chemistry, Department of Chemistry, Aristotle University of Thessaloniki, 54124 Thessaloniki, Greece

**Keywords:** fenamates, transition metal complexes, cobalt(II) complexes, antioxidant activity, antibacterial activity, interaction with DNA, affinity for albumins

## Abstract

A nitro-derivative of fenamic acid (4′–nitro–fenamic acid) was synthesized and used as ligand for the synthesis of four Co(II) complexes in the absence or presence of the *N*,*N*′-donors 2,2′–bipyridylamine, 1,10–phenanthroline and 2,9–dimethyl–1,10–phenanthroline. The characterization of the resultant complexes was performed with diverse techniques (elemental analysis, molar conductivity measurements, IR and UV-vis spectroscopy, single-crystal X-ray crystallography). The biological evaluation of the compounds encompassed (i) antioxidant activity via hydrogen peroxide (H_2_O_2_) reduction and free radical scavenging; (ii) antimicrobial screening against two Gram-positive and two Gram-negative bacterial strains; (iii) interactions with calf-thymus (CT) DNA; (iv) cleavage of supercoiled pBR322 plasmid DNA (pDNA), in the dark or under UVA/UVB/visible light irradiation; and (v) binding affinity towards bovine and human serum albumins. The antioxidant activity of the compounds against 2,2′–azinobis–(3–ethylbenzothiazoline–6–sulfonic acid) radicals and H_2_O_2_ is significant, especially in the case of H_2_O_2_. The complexes exhibit adequate antimicrobial activity against the strains tested. The complexes interact with CT DNA through intercalation with binding constants reaching a magnitude of 10^6^ M^−1^. The compounds have a significantly enhanced pDNA-cleavage ability under irradiation, showing promising potential as photodynamic therapeutic agents. All compounds can bind tightly and reversibly to both albumins tested.

## 1. Introduction

Cobalt, being an essential trace element for humans, plays a pivotal role in medicine, mainly because of its key presence in cobalamin (vitamin B12), an important nutrient that is involved in a plethora of biological processes such as DNA synthesis, the formation of red blood cells or a variety of neurological functions. Conversely, studies have shown a link between cobalt deficiency and serious health issues, e.g., chronic inflammation (connected to several cancer types [1,2]), anorexia or anemia [3,4]. Moreover, a significant amount of current research has been focused on the synthesis and investigation of novel cobalt complexes that exhibit promising pharmacological properties, including anti-inflammatory, antiproliferative, antioxidant, antimicrobial and antiviral activities [5,6,7,8].

Non-steroidal anti-inflammatory drugs (NSAIDs) constitute a widely used weapon in the arsenal of modern medicine based on their anti-inflammatory, analgesic and antipyretic properties, leading to a global NSAID market size of approximately USD 15 billion (in 2019) which is projected to significantly enhance [9] making up ~10% of annually prescribed medication [10,11]. Commonly employed for alleviating inflammation, fever and pain, they play a critical role in addressing chronic disorders, such as arthritis, and acute injuries. Their therapeutic effects arise from the suppression of cyclooxygenase (COX) enzymes, which catalyze the production of prostaglandins, the key mediators of inflammatory and pain pathways. Emerging evidence highlights their expanded pharmacological potential, including applications in cancer prevention, mitigation of Alzheimer’s disease progression, antimicrobial activity against bacterial and viral pathogens and adjunct roles in the management of depressive disorders [5,12,13,14,15]. However, gastrointestinal or cardiovascular side effects limit the possible therapeutic efficacy of NSAIDs usage, indicating the necessity of further research into their mechanisms of action and the importance of developing safer alternatives with milder side effects.

**Figure 1:**
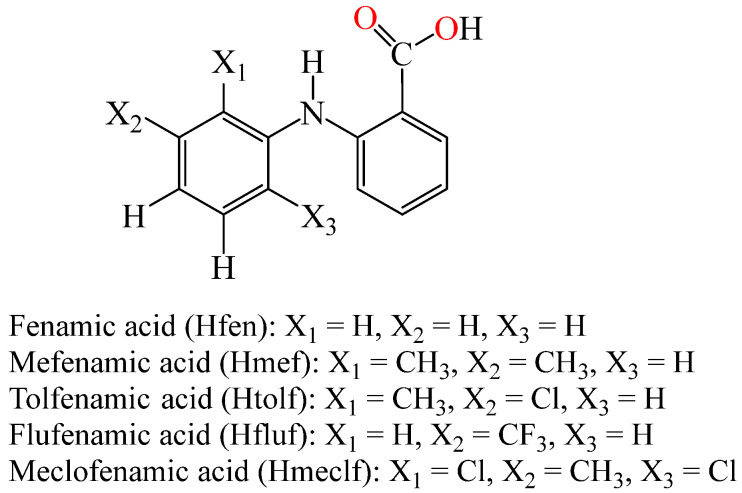
The syntax formulas of the commercially available fenamates: fenamic acid (Hfen) and its derivatives: mefenamic acid (Hmef), tolfenamic acid (Htolf), flufenamic acid (Hfluf) and meclofenamic acid (Hmeclf).

Fenamic acid (Hfen) is a derivative of anthranilic acid and constitutes the foundational scaffold for a significant family of NSAIDs known as fenamates, which are commercially available with mefenamic acid (Hmef), tolfenamic acid (Htolf), flufenamic acid (Hfluf) and meclofenamic acid (Hmeclf) (Figure 1) being the most characteristic members. Their differentiation stems from the presence of alkyl or halogen substituents at the aromatic ring positions. Widely employed, these compounds exhibit a broad spectrum of pharmacological properties underpinning their extensive use in clinical practice as anti-inflammatory, analgesic or antipyretic agents. A growing body of evidence suggests that their use can be diverted from their mainstream use, to help the increasingly important fight against bacteria, neurodegenerative diseases and cancer [15,16,17,18].

**Figure 2:**
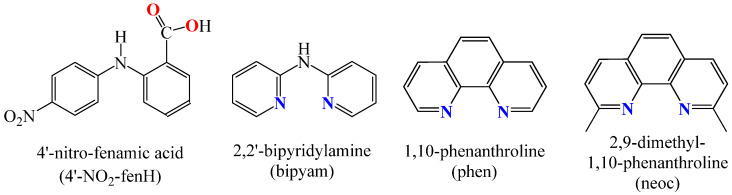
The syntax formulas of 4′–nitrofenamic acid (4′–NO_2_–fenH), 2,2′–bipyridylamine (bipyam), 1,10–phenanthroline (phen) and 2,9–dimethyl–1,10–phenanthroline (neoc).

The increased biological activity of fenamates prompts us to synthesize a nitro-derivative of fenamic acid, namely 4′–nitro–fenamic acid (4′–NO_2_–fenH, Figure 2). The existence of a nitro group on a drug candidate offers, in many cases, stability, solubility and enhanced interaction with molecular targets due to its high polar and electron-withdrawing effects. In addition, it provides good absorption under UVA or visible light that in some cases cause the photoactivation of molecular oxygen that leads to promising photodynamic activity [19,20,21]. Nevertheless, nitro-containing drugs come with skepticism over their therapeutic effect and toxicity ratio, which needs to be carefully balanced, as the existence of the nitro group is not the only factor to challenge toxicity [22]. Besides their equivocal behavior, a number of nitro-drugs exist in the market, like nitrofurantoin, which is used for the treatment of lower urinary tract infections and the natural products chloramphenicol and pyrrolomycins, which are known antibacterial agents [23]. In other drugs, the nitro group benefits activity due to its bioreductive potential and/or stabilization via H-bond interactions [24,25]. It seems also important to mention that in some prodrug cases, the nitro group was found essential for DNA binding activity [20,24]. However, concerning the binding affinity to bovine serum albumin of several *N*-phenyl anthranilic acids, the existence of the nitro group in the case of 4′–NO_2_–fenH (Figure 2) lowered the binding affinity [26]. Thus, it seems imperative to check not only the ligand itself, but the influence of the loaded metal and the co-ligands on this particular fenamic acid towards biological activity.

As a continuation of our research project regarding the synthesis, the characterization and the biological evaluation of a series of Co(II)–NSAID complexes [27,28,29,30,31,32,33], four novel Co(II) complexes of 4′–NO_2_–fenH were synthesized in the absence or presence of the *N*,*N*′-donors 2,2′–bipyridylamine (bipyam), 1,10–phenanthroline (phen) and 2,9–dimethyl–1,10–phenanthroline (neocuproine, neoc) as co-ligands, namely [Co(4′–NO_2_–fen)_2_(MeOH)_4_] (complex **1**), [Co(4′–NO_2_–fen)_2_(bipyam)] (complex **2**), [Co(4′–NO_2_–fen)_2_(phen)] (complex **3**) and [Co_2_(4′–NO_2_–fen)_2_(μ–CH_3_O)_2_(neoc)_2_] (complex **4**). The compounds were characterized with physicochemical (elemental analysis and molar conductivity measurements) and spectroscopic techniques (IR and UV-vis spectroscopy), and the molecular structure of complex **4** was determined with single-crystal X-ray crystallography.

The evaluation of the biological profile of the synthesized compounds focuses on their antioxidant and antimicrobial efficacy, alongside their interaction with biomolecules, i.e., DNA and albumins. Standardized assays were used to assess the antioxidant activity of the compounds, namely the scavenging of 1,1–diphenyl–picrylhydrazyl (DPPH) and 2,2′–azinobis–(3–ethylbenzothiazoline–6–sulfonic acid) (ABTS) free radicals, and the reduction of hydrogen peroxide. Antimicrobial activity was assayed by determining the minimum inhibitory concentration (MIC) against two Gram-negative pathogens *Escherichia coli XL1* (*E. coli*) and *Xanthomonas campestris ATCC 33013* (*X. campestris*), as well as two Gram-positive strains *Staphylococcus aureus ATCC 29213* (*S. aureus*) and *Bacillus subtilis ATCC 6633* (*B. subtilis*).

The interaction of the compounds with calf-thymus (CT) DNA was monitored with UV-vis spectroscopy, viscosity measurements and fluorescence emission spectroscopy aiming to calculate the corresponding DNA-binding constant and to assess the DNA-interaction mode, the competition with ethidium bromide (EB) and the effect of temperature on this interaction. In the context of investigating the nuclease-like activity of the compounds, their interaction with supercoiled circular pBR322 plasmid DNA (pDNA) was studied using gel electrophoresis to assess the pDNA-cleavage ability and the effect of irradiation with UVA, UVB or visible light on the cleavage. Fluorescence quenching studies were employed to determine the affinity of the compounds for bovine (BSA) and human serum albumins (HSAs). Within this context, the corresponding albumin-binding constants of the compounds were calculated and the main albumin-binding sites were determined through competitive displacement experiments using site-specific probes.

## 2. Results and Discussion

### 2.1. The Synthesis and Characterization of the Compounds

**Scheme 1:**
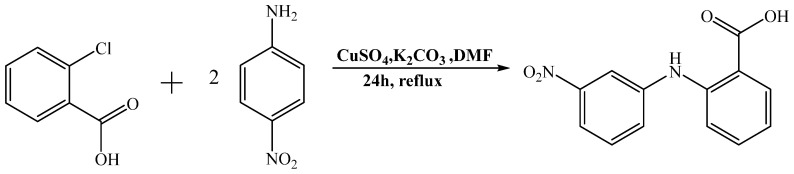
Synthesis of compound 4′-NO_2_-fenH using 2-chlorobenzoic acid (1 eq), 4-nitroaniline (2 eq), CuSO_4_ (catalyst), K_2_CO_3_ (0.5 eq), DMF, reflux 24 h, 86% yield.

The synthesis of 4′-NO_2_-fenH (Scheme 1) was loosely based on previously reported procedures [34,35] that were modified to achieve increased yield and easier synthesis (e.g., inert atmosphere was used previously, but no significant differences were noticed in the purity of product, and the yield was similar or increased). The precipitate, after acidification of the reaction and stirring for 24 h, was recrystallized to give the pure compound 4′–NO_2_–fenH. The resultant compound 4′–NO_2_–fenH was characterized by ^1^H NMR and IR spectroscopies (Figures S1 and S2, respectively) and the obtained ^1^H NMR spectral data are in correlation with the literature [36].

Complexes **1**–**4** were synthesized in relatively high yield via the aerobic reaction of a methanolic solution (for complexes **1** and **2**) or a 1:1 MeOH–dichloromethane mixture (for complexes **3** and **4**) of deprotonated 4′–NO_2_–fenamic acid with CoCl_2_·6H_2_O in the presence or absence of the corresponding *N*,*N*′-donor. More specifically, for complex **1**, a 1:2 Co^2+^:(4′–NO_2_–fen^−1^) ratio was used, and a 1:2:1 Co^2+^:(4′–NO_2_–fen^−1^):(*N*,*N*′-donor) was applied for the synthesis of complexes **2**–**4**.

The resultant complexes were characterized using IR and UV-vis spectroscopy, while the crystal structure of complex **4** was determined with single-crystal X-ray crystallography. All the complexes are stable in air, soluble in DMSO, DMF, MeOH and EtOH and partially soluble in H_2_O and CH_3_CN. Molar conductivity (Λ_Μ_) for complexes **1**–**4** was measured in DMSO solution (1 mM) and the values fall into the range 9–18 S∙cm^2^∙mol^−1^ showing that the complexes are non-electrolytes in DMSO [37] and keep their integrity.

IR spectroscopy was employed to investigate the mode of its coordination of the deprotonated 4′–NO_2_–fen^−^ ligands to the Co(II) ion as well as the presence of the nitrogen-donor co-ligands (Figure S2). Two characteristic bands of the carboxylato groups of complexes **1**–**4** were investigated. They are attributed to the antisymmetric (*v*_asym_(COO)) and symmetric (*v*_sym_(COO)) stretching vibrations and were observed in the regions 1584–1593 cm^−1^ and 1361–1395 cm^−1^, respectively. The value of Δ*v* (COO) is in the range 189–232 cm^−1^, suggesting an asymmetric coordination of the carboxylato group of the 4′–NO_2_–fen^−^ ligands [38,39]. Additionally, bands attributable to out-of-plane ρ(C–H) vibration of the *N*,*N*′-donors (ρ(C–H)_bipyam_: 770 cm^−1^ in **2**, ρ(C–H)_phen_: 725 cm^−1^ in **3** and ρ(C–H)_neoc_: 733 cm^−1^ in **2**, verified their existence in the complex [38].

In the visible region of the UV-vis spectra of the complexes recorded in DMSO solution, the bands observed in region 480–670 nm can be attributed to d–d transitions, characteristic of octahedral Co(II) complexes [40]. Furthermore, a band in the region 391–427 nm can be attributed to a charge-transfer transition, while one or two bands in the range 269–370 nm can be attributed to intraligand transitions.

### 2.2. Structure of the Compounds

Among the four Co(II) complexes studied herein, single-crystals suitable for X-ray crystallography structural determination were obtained only for complex **4**. Therefore, the structural characterization of complexes **1**–**3** was performed based on derived experimental data and after comparison with the literature.

#### 2.2.1. Crystal Structure of [Co_2_(4′–NO_2_–fen)_2_(μ–CH_3_O)_2_(neoc)_2_] (Complex **4**)

**Figure 3:**
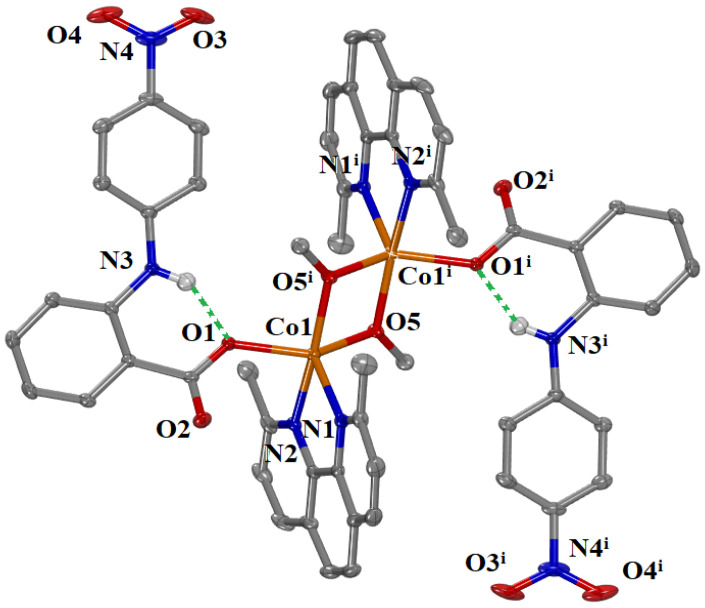
Molecular structure of complex **4** [Co_2_(4′–NO_2_–fen)_2_(μ–CH_3_O)_2_(neoc)_2_] (symmetry code: (i) −*x* + 1, −*y* + 1, −*z* + 1). Aromatic and methyl hydrogen atoms are omitted for clarity reasons. The green dashed lines represent the intraligand hydrogen bonds. Carbon atoms are shown in grey.

The molecular structure of complex **4** is shown in Figure 3, and selected bond lengths and angles are summarized in Table 1. The complex crystallized in the triclinic crystal system and *P*ī space group (Table S1).

It is a centrosymmetric dinuclear Co(II) complex having an inversion center in the middle of the distance of two Co(II) ions. The Co(II) ions are bridged with two methoxo oxygen atoms. The interatomic Co1…Co1^i^ distance is 3.122 Å and lies close to the distance range found for dinuclear Co(II) complexes with two methoxo bridges (2.923–3.0585 Å) [41,42,43]. Complex **4** is among the few reported examples of dinuclear Co(II) complexes containing two methoxo bridges [41,42,44], since most of the reported methoxo-bridged cobalt(II) complexes are of higher nuclearity [43,45,46,47,48,49,50,51,52,53]. The Co(II) ions are pentacoordinate and each coordination contains the two bridging methoxy oxygen atoms O5 and O5^i^, one oxygen atom O1 from the monodentate 4′–NO_2_–fen^−^ ligand and two nitrogen atoms N1 and N2 from the bidentate neoc ligand. The value of the trigonality index is (τ_5_ = (φ_1_ − φ_2_)/60°, where φ_1_ and φ_2_ are the largest angles in the coordination sphere; τ_5_ = 0 is found for a perfect square pyramid and τ_5_ = 1 for a perfect trigonal bipyramid) [54] is τ_5_ = (161.19–140.41)/60 = 0.35, and indicates a significantly distorted pyramidal geometry around the Co(II) ion.

Intraligand hydrogen bonds (given as green dashed lines in Figure 3) are developed between the coordinated carboxylato oxygen O1 and the amino hydrogen H31 of 4′–NO_2_–fen^−^ ligands (N3—H31 = 0.85 Å, H31···O1 = 1.89 Å, N3···O1 = 2.596(4) Å, N3—H31··· O1 = 140º) contributing to further stabilization of the structure of complex **4**.

#### 2.2.2. Proposed Structures for Complexes **1**–**3**

Based on the experimental data (molar conductivity measurements, IR and UV-vis spectroscopies) and after a comparison with the literature, the structures for complexes **1**–**3** can be proposed. All complexes are non-electrolytes and contain deprotonated 4′–NO_2_–fen^−^ and the corresponding nitrogen-donor ligands (for complexes **2** and **3**).

Complex **1** is a mononuclear octahedral Co(II) compound where the 4′–NO_2_–fen^−^ ligands are monodentately coordinated. It is expected to have a CoO_6_ coordination sphere with two of the six oxygen atoms originating from the carboxylato groups of two 4′–NO_2_–fen^−^ ligands and the other four oxygen atoms from four methanol ligands (Figure S3). Such an arrangement is similar to that reported for complexes [Co(mef)_2_(MeOH)_4_] [28], [Co(Hdifl)_2_(MeOH)_4_] and [Co(nif)_2_(MeOH)_4_] [32] (Hmef, H_2_difl, and Hnif are the NSAIDs mefenamic acid, diflunisal and niflumic acid, respectively).

Complexes **2** and **3** are mononuclear compounds where the 4′–NO_2_–fen^−^ ligands are coordinated to Co(II) ion in an asymmetric fashion. The coordination sphere of the six-coordinate Co(II) ion consists of four oxygen atoms from two 4′–NO_2_–fen^−^ ligands and two nitrogen atoms from bipyam and phen ligands (Figure S3). The complexes are expected to be isostructural with a series of Co(II)–NSAID complexes bearing the formula [Co(NSAID)_2_(*N*,*N*′-donor)], such as [Co(tolf)_2_(neoc)], [Co(mef)_2_(neoc)], [Co(nap)_2_(neoc)] [29], [Co(dicl)_2_(bipy)], [Co(dicl)_2_(bipyam)] and [Co(dicl)_2_(phen)] [30], [Co(fluf)_2_(bipyam)] and [Co(mef)_2_(bipyam)] [32], [Co(tolf)_2_(bipyam)] [33], and [Co(fen)_2_(neoc)] [55] (Hdicl and Hnap are the NSAIDs diclofenac and naproxen, respectively).

### 2.3. The Antioxidant Activity of the Compounds

Free radicals are reactive species containing unpaired electrons. The transfer of these unpaired electrons to different molecules may trigger chain free radicals are reactive species containing unpaired electrons. The transfer of these unpaired electrons to different molecules may trigger chain reactions that lead to swelling, tissue damage or even cancer [56]. NSAIDs can combat this phenomenon, either by neutralizing free radicals or by preventing their formation. Given this, novel compounds with antioxidant properties can play a vital role in managing inflammation and lead to the development of new pharmaceuticals. In this study, the antioxidant activity of the synthesized compounds was assessed based on their ability to scavenge DPPH and ABTS radicals and to reduce H_2_O_2_, and the results (Table 2) were compared to the standard antioxidants nordihydroguaiaretic acid (NDGA), butylated hydroxytoluene (BHT), 6–hydroxy–2,5,7,8–tetramethylchroman–2–carboxylic acid (trolox) and L-ascorbic acid [57,58].

Antioxidant compounds able to neutralize DPPH radicals are increasingly recognized due to their capability of combating cancer, slowing down the aging process and regulating inflammation. As a result, they could potentially offer protection against rheumatoid arthritis and inflammation. DPPH is a stable free radical exhibiting an intense absorption band at 517 nm [59]. In this experiment, NDGA and BHT were used as reference compounds and measurements took place at 30 min and 60 min. As shown in Table 2, none of the compounds (4′–NO_2_–fenH and its complexes **1**–**4**) were active against DPPH radicals. Such low scavenging activity of DPPH radicals is rather common for metal–NSAID complexes [27,28,29,30,31,32,33,60,61,62,63,64].

The ABTS-scavenging was measured through the discoloration of a dark green solution containing the cationic radical ABTS^•+^, which was induced from the oxidation derived from hydrogen-donating antioxidants, either complexes or standards, and is often considered a marker of total oxidation capacity [65]. As shown in Table 2, the complexes present moderate ABTS-scavenging ability, showing an enhancement of the activity of 4′–NO_2_–fenH (which is inactive in its free form). Such an increase in activity upon coordination is expected for metal–NSAID complexes [27,28,29,30,31,32,33,60,61,62,63,64]. However, the activity of the complexes is significantly lower than that of the reference compound trolox.

Harmful hydroxyl radicals are released when hydrogen peroxide interacts with transition metal ions, leading to oxidative stress. Consequently, both oxidative stress and the production of reactive oxygen species could be reduced by ^•^OH scavengers and H_2_O_2_ reducing agents [66]. As presented in Table 2, complexes **1**–**4** are more active (H_2_O_2_% = 69.48–77.59%) when interacting with H_2_O_2_ than 4′–NO_2_–fenH (H_2_O_2_% = 66.46%). Furthermore, all tested complexes demonstrate superior H_2_O_2_ reduction comparatively to L-ascorbic acid, which was used as the reference compound. Complex **2** exhibited the best activity among all the compounds tested herein (H_2_O_2_% = 77.59 ± 0.28%). The behavior of the compounds towards H_2_O_2_ is within the range reported for other metal–NSAID complexes [29,31,60,62,64,67,68].

In total, the compounds were practically inactive towards DPPH radicals and moderately active towards ABTS radicals. All compounds studied herein were more active towards H_2_O_2_ than the reference compound L–ascorbic acid with complex **3** showing the best activity.

### 2.4. The Antibacterial Activity of the Compounds

In order to evaluate the antimicrobial activity of the synthesized 4′–NO_2_–fenH and its Co(II) complexes **1**–**4**, two Gram-positive (*B. subtilis* and *S. aureus*) and two Gram-negative (*X. campestris* and *E. coli*) strains were used. In general, the increased antimicrobial activity of the complexes is attributed to five main factors: (i) the chelation effect of the ligands, (ii) the chemical nature of the ligands, (iii) the nature and the nuclearity of the metal center, (iv) the total charge of the complex and (v) the presence and nature of counter ions [69,70].

According to the results shown in Table 3, the MIC values of the compounds fall within the range of 12.5–50 μg/mL showing a moderate-to-significant activity. More specifically, 4′–NO_2_–fenH has MIC values in the range 12.5–25 μg/mL (48–97 μM) against the bacteria tested and complexes **1**–**4** present MIC values in the range 12.5–50 μg/mL (11–71 μM). When considering the MIC values in the molar scale, it is quite clear that the coordination of 4′–NO_2_–fen^−^ ligand to Co(II) greatly enhances the antibacterial activity. In addition, the presence of *N*,*N*′-donor co-ligands might also affect the antimicrobial activity of the compounds. Complexes **3** and **4** containing phen and neoc as *N*,*N*′-donors show an increased activity compared to complexes **1** (absence of *N*,*N*′-donor) and **2** (containing bipyam), though further studies and comparison should be conducted to pinpoint the specific mechanism.

### 2.5. Interaction of the Compounds with CT DNA

Complexes of biological relevance can interact with linear DNA either covalently (forming a bond with a nitrogen atom of the DNA-base) or non-covalently (via intercalation (π–π stacking interactions are observed), through groove-binding (van der Waals forces or hydrogen-bonds are developed), or electrostatically (via Coulomb forces)), or/and can induce cleavage of DNA [71]. The affinity of the compounds (i.e., 4′–NO_2_–fenH and complexes **1**–**4**) for CT DNA was investigated using UV-vis spectroscopy, viscosity measurements and by studying their ability to displace EB on the EB–DNA complex as monitored with fluorescence emission spectroscopy.

#### 2.5.1. The Study of the Interaction of the Compounds with CT DNA Using UV-Vis Spectroscopy

UV-vis spectroscopy was employed to determine the interaction mode of the compounds (4′–NO_2_–fenH and its complexes **1**–**4**) with DNA and calculate the DNA-binding constant (K_b_). For this reason, the UV-vis spectra of the compounds (2 × 10^−5^–10^−4^ Μ) were recorded in the presence of incremental additions of CT DNA solution (Figure S4), and the changes (in absorbance and wavelength) of the initial bands were monitored. Most of these intraligand bands exhibited hypochromism in the presence of CT DNA (Table 4), which might be evidence of intercalation, which arises from the π–π stacking interaction between the aromatic chromophore of the compound and the DNA-base pairs [72]. However, a definite conclusion cannot be reached from such spectroscopic data, but the interaction mode could be further corroborated by viscosity measurements.

The K_b_ values of the compounds were calculated with the Wolfe–Shimer equation (Equation (S1)) [73] and the corresponding plots of [DNA]/(ε_A_–ε_F_) vs. [DNA] (Figure S5). It is interesting to note that compared to the commercially available fenamates mefenamic acid, tolfenamic acid and flufenamic acid with K_b_ values 1.05 × 10^5^, 5.00 × 10^4^ and 2.70 × 10^5^ M^−1^ [28,33,74], respectively, 4′–NO_2_–fenamic acid bears much higher K_b_ (1.62 × 10^6^ M^−1^). Moreover, in comparison to the classic DNA intercalator EB (K_b_ = 1.23 × 10^5^ M^−1^) [75], 4′–NO_2_–fenH and complexes **3**–**4** demonstrated higher K_b_ values, proving tighter binding to DNA. In addition, the K_b_ values of complexes **1**–**4** (Table 4) were found to be in the range 7.20 × 10^4^–2.06 × 10^6^ M^−1^, and are consistent with the K_b_ values of similar Co(II)–NSAID complexes reported in the literature [27,28,29,30,31,32,33].

#### 2.5.2. The Effect of Temperature on the Interaction of the Compounds with CT DNA

In order to further clarify the interaction mode of the compounds with CT DNA, the effect of the temperature of this interaction was also investigated. Within this context, the thermal denaturation of DNA in the presence of the compounds was studied with UV-vis spectroscopy in order to calculate (via a sigmoidal fitting of the experimental data) the DNA-melting temperature (*T*_m_), i.e., the temperature at which half of double-stranded DNA is denatured (“melted”) to single-stranded DNA.

**Figure 4:**
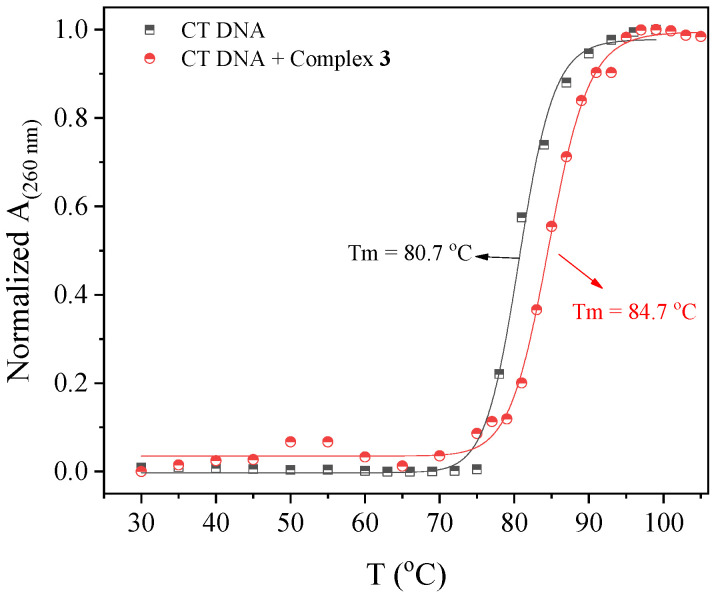
Thermal melting profile of CT DNA in the absence or the presence (compound–DNA ratio = 1:50) of complex **3**.

The UV spectra of a CT DNA solution (1.25 × 10^−4^ M) in the presence of the compounds in a 1:50 compound–DNA ratio were recorded for increasing temperatures and the changes in the DNA UV band with λ_max_ at 260 nm were monitored vs. temperature (Figure S6). In the presence of 4′–NO_2_–fenH and complexes **1** and **3**, an increase in *T*_m_ of CT DNA from 80.7 °C to 83.7–84.7 °C (Figure 4, Table 4) was observed (i.e., Δ*T*_m_ = 3–4 °C) revealing the stabilization of CT DNA from the compounds as a result from an intercalative interaction [76,77,78], while complexes **2** and **4** seemed to induce destabilization of the CT DNA duplex since a negative ΔT_m_ was found [78,79].

#### 2.5.3. The Study of Interaction of the Compounds with CT DNA with Viscosity Measurements

Viscometry is a technique used to determine the mode of interaction between complexes and DNA. In this method, the changes in the DNA-viscosity (it is sensitive to length changes in the DNA chain) are monitored upon the addition of increasing amounts of the compounds into the DNA solution. An external interaction of the compound with DNA (electrostatic interaction or groove-binding) will not significantly affect the length of the DNA-chain, resulting in a slight bending of the double helix, and subsequently, negligible changes or a slight decrease in viscosity will be observed. In the case of classic intercalation, the intercalating compound will cause a further separation of the nitrogen DNA-bases for its accommodation, thus leading to an increase in the chain length and consequently higher viscosity. Furthermore, the cleavage of DNA will produce shorter fragments, and the DNA-viscosity will decrease significantly [80].

**Figure 5:**
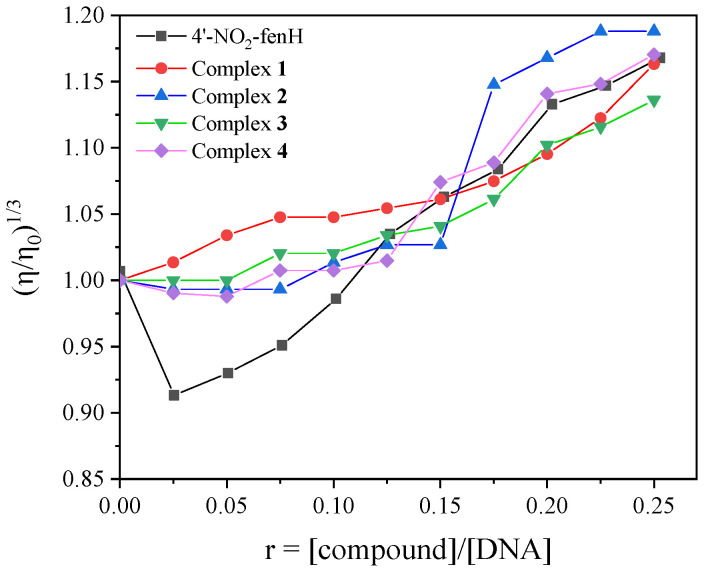
Relative viscosity (η/η_0_)^1/3^ of CT DNA (0.1 mM) in buffer solution (150 mM NaCl and 15 mM trisodium citrate at pH 7.0) in the presence of the compounds at increasing amounts (r = [compound]/[DNA]).

In this study, the changes in the viscosity of the DNA solution (0.1 mM) were monitored in the presence of increasing amounts (Figure 5) of the compounds (up to r = [compound]/[DNA] = 0.25). In particular for 4′–NO_2_–fenH, a slight decrease in viscosity was initially (up to r = 0.1) observed, suggesting a possible external interaction with the DNA chain in order to approach closer to DNA, while further additions of 4′–NO_2_–fenH resulted in increased viscosity. The increase in relative DNA viscosity observed for all compounds indicates that classic intercalation occurs upon their interaction with CT DNA.

#### 2.5.4. Study of EB-Competition of the Compounds upon Interaction with CT DNA

EB is a fluorescent indicator of intercalation. EB interacts with the DNA double helix through the intercalation of its planar phenanthridine ring in-between two adjacent bases of the DNA double helix forming a soluble adduct which exhibits an intense fluorescence emission band with λ_max,emission_ in the range 592–594 nm, with λ_excitation_ at 540 nm [81]. Changes in this emission band were monitored to indirectly study the interaction of CT DNA with compounds upon their addition. If the added compound binds to the DNA as strongly as EB, then an intense decrease on the EB–DNA emission band will be observed. On the other hand, if the compound does not intercalate to DNA, a slight or negligible decrease in fluorescent emission is detected, reflecting its inability to displace EB [81].

**Figure 6:**
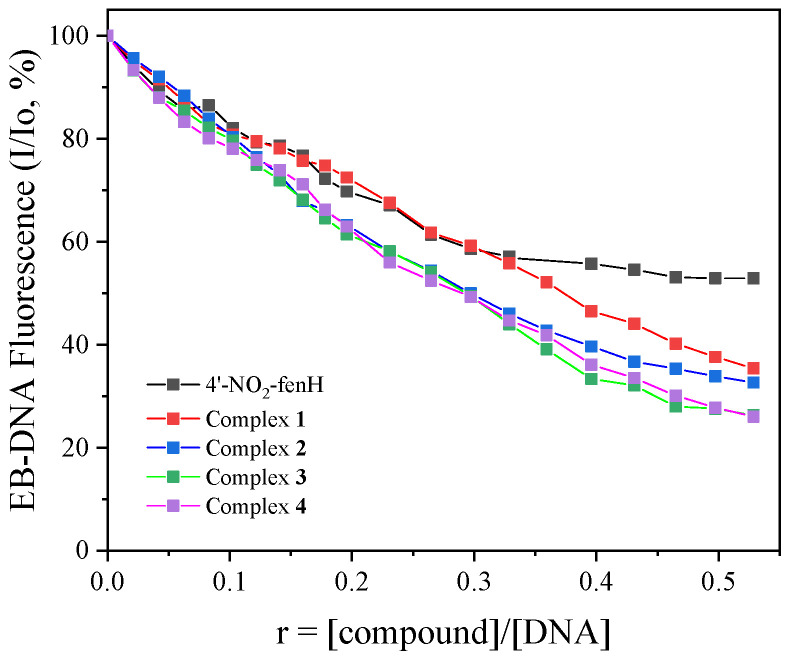
Plot of relative EB–DNA fluorescence emission intensity at λ_emission_ = 594 nm (I/Io, %) vs. r (r = [compound]/[DNA]) in the presence of 4′–NO_2_–fenH and its complexes **1**–**4** (up to 52.9% of the initial EB–DNA fluorescence for 4′–NO_2_–fenH, 35.4% for complex **1**, 32.6% for complex **2**, 26.3% for complex **3** and 26.0% for complex **4**).

For the current study, the EB–DNA adduct was prepared in situ after 1-h pretreatment of EB (40 μΜ) and CT DNA (40 μΜ) in buffer solution (sodium chloride 150 mM and sodium citrate 15 mM at pH = 7). The compounds (which did not exhibit any emission bands under excitation at 540 nm) were added incrementally into such a solution and the changes in the fluorescence emission spectra were monitored (Figure S7). More specifically, the presence of the compounds induced an intensive decrease in the fluorescence emission band at 594 nm (up to 74% of the initial fluorescence intensity, Figure 6, Table 5) which can be assigned to displacement of EB from the EB–DNA adduct by the compounds [81].

Furthermore, the Stern–Volmer constants (K_SV_) that represent the ability of the compounds to induce quenching were calculated with the Stern–Volmer equation (Equation (S2)) and the corresponding Stern–Volmer plots I/I_0_ vs. [Q] (Figure S8). All compounds under study demonstrate relatively high K_SV_ values. The corresponding quenching constants (K_q_) were calculated with Equation (S3) using the value of 23 ns as the fluorescence lifetime (τ_0_) of EB–DNA adduct [82]. The K_q_ values of all compounds are much higher than 10^10^ M^−1^s^−1^, indicating a static quenching mechanism, which suggests the formation of a new adduct between the compounds and DNA [81], thus verifying the displacement of EB and indirectly showing an intercalative interaction of the compounds with CT DNA.

### 2.6. The Cleavage and Photocleavage of pDNA Induced from the Compounds

The cleavage of pBR322 plasmid DNA was evaluated both in the presence and absence of irradiation in order to further explore the interaction of the synthesized compounds with DNA and their possible biological efficacy. To achieve this, the compounds (500 µM) were mixed with pDNA in a Tris buffer solution (25 μM, pH 6.8), ensuring that the final DMSO concentration did not exceed 10% *v*/*v*. Following incubation of the samples at 37 °C, the impact of the compounds on pDNA was assessed using gel electrophoresis on 1% agarose gels stained with EB. Irradiation at 312 nm (UVB for 30 min), at 365 nm (UVA, for 120 min) or visible light (for 120 min) was also applied to further investigate the possibility of photocleavage activity of the compounds. The interaction of the compounds with pDNA resulted in three different forms of pDNA: Form I (supercoiled pDNA), Form II (relaxed pDNA) induced from single-stranded (ss) damage, and Form III (linear pDNA) induced from double-stranded (ds) damage. The extent of pDNA damage from each compound was assessed by calculating the percentage of ss% and ds% using Equations (S4) and (S5).

**Figure 7:**
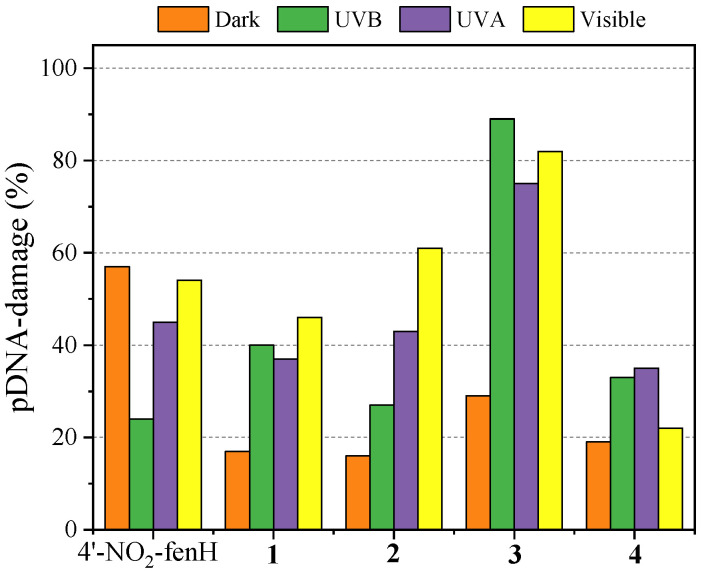
Overall percentage of damage induced by the compounds to pDNA (pDNA damage, in %) in the absence (dark) or presence of UVB, UVA and visible irradiation.

The reaction mixtures of pDNA and the compounds were incubated in the dark for 150 min and were analyzed by 1% agarose gel electrophoresis with EB-staining (Figure S9). In the absence of irradiation, the compounds (500 μM) induced a low-to-moderate percentage of ss breaks, resulting in relaxed circular DNA (Form II). Among the compounds studied herein, only 4′–NO_2_–fenH induced ds breaks, resulting in linear pDNA (Form III), leading to a total pDNA damage (57%) and being the most active compound in the absence of light (Figure 7).

Irradiation increased the activity of all compounds, resulting in higher pDNA damage. After exposure to UVB irradiation for 30 min, all the compounds (except for 4′–NO_2_–fenH) showed increased activity compared to the absence of irradiation due to ss breaks (Figure S10), with compound **3** (the only compound that induced additional ds damage) exhibiting the highest pDNA damage of 89% (highest among all compounds regardless the presence of irradiation, Figure 7).

Exposure to UVA irradiation for 120 min increased also the activity of the compounds, leading to higher pDNA damage, with generally more efficient photocleavage, with compound **3** exhibiting again the highest activity (Figure 7). Complexes **2**, **3** and 4′–NO_2_–fenH also caused ds breaks leading to the formation of linear pDNA (Form III) (Figure S11). It is noteworthy that in the case of 4′–NO_2_–fenH and compound **3**, a new band was formed, exhibiting a slower electrophoretic mobility compared to that of Form II of pDNA (Figure S11), possibly attributed to pDNA fragments of higher molecular weight [83,84].

The highest activity for all compounds (except compound **3**, that had similar activity under UVB and visible light irradiation and compound **4**) was shown after exposure to visible light for 120 min (Figure 7). That can be verified by the formation of linear pDNA (Form III) in all cases (except for compound **4**) apart from only relaxed circular pDNA (Figure S12), leading us to the conclusion that exposure to visible light effectively increased the cleavage ability of the compounds, resulting in both ss and ds breaks of pDNA.

Based on the evidence presented, irradiation energy and exposure time affect the total photocleavage activity of evaluated compounds. Except for 4′–NO_2_–fenH, irradiation of any wavelength increased the photocleavage activity of all compounds. This can be attributed to the presence of the Co(II) center in the complexes, which increases the absorbance of the compounds. Specifically, this assumption can be verified by the highest increase in photocleavage activity under visible light irradiation and the fact that the synthesized compounds exhibit high absorbance at approximately 400 nm in their UV-vis spectra. This differentiated (photo)cleave behavior leads us to suggest a potential efficacy of the synthesized compounds as chemotherapeutic agents. Despite the multifaceted nature of pDNA photocleavage—being influenced by parameters such as irradiation time, photon energy, DNA-binding affinity and structural diversity—all compounds can be regarded as photoreactive.

### 2.7. Interaction of the Compounds with Serum Albumins

#### 2.7.1. Affinity of the Compounds for Serum Albumins

Serum albumins (SAs) are the most abundant proteins in blood plasma performing various functions such as maintaining osmotic pressure and transporting numerous exogenous and endogenous substances [85]. The binding of drugs to SAs may affect drug delivery to their biological targets and their disposition. Among SAs, bovine serum albumin (BSA) has been extensively studied due to its structural homology with human serum albumin (HSA) and its high stability [86]. The biological studies of SAs rely on their fluorescence emission which arises from the single tryptophan residue in HSA (Trp–214) and two tryptophan residues in the BSA (Trp–212 and Trp–134) [87]. In the present study, the interaction of the compounds (4′–NO_2_–fenH and its complexes **1**–**4)** with BSA and HSA was investigated via fluorescence emission spectroscopy.

**Figure 8:**
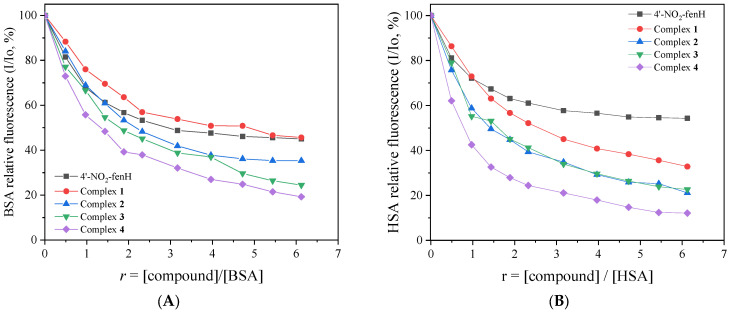
(**A**) Plot of % relative fluorescence intensity of BSA at λ_em_ = 345 nm (I/Io, %) vs. *r* (*r* = [compound]/[BSA]) for 4′–NO_2_–fenH and its complexes **1**–**4** (up to 45.0% of the initial BSA fluorescence for 4′–NO_2_–fenH, 45.6% for complex **1**, 35.3% for complex **2,** 24.4% for complex **3** and 19.2% for complex **4**). (**B**) Plot of % relative fluorescence intensity of HSA at λ_em_ = 341 nm (I/Io, %) vs. *r* (*r* = [compound]/[HSA]) for 4′–NO_2_–fenH and its complexes **1**–**4** (up to 54.3% of the initial HSA fluorescence for 4′–NO_2_–fenH, 32.8% for complex **1**, 21.1% for complex **2**, 22.7% for complex **3** and 12.1% for complex **4**).

The interaction between complexes **1**–**4** and albumins was investigated by recording the tryptophan fluorescence emission spectra and monitoring the quenching induced by incremental amounts of the complexes. For these experiments, albumins (3 mM) were prepared in buffer solution (150 mM NaCl and 15 mM trisodium citrate at pH = 7) and their fluorescence emission spectra were recorded in the range 300—500 nm for λ_excitation_ = 295 nm. Upon incremental addition of the compounds (Figures S13 and S14), a significant decrease in the intensity of the corresponding emission band at λ_max,emission_ = 341 nm and 345 nm for HSA and BSA, respectively, was observed (up to 87% of the initial fluorescence, Figure 8). The observed fluorescence emission quenching might be a result of a change in the spatial conformation of the protein and specifically of rearrangement of its secondary structure, as a result of SA–complex interactions [81]. To quantify this interaction, the fluorescence emission spectra (λ_excitation_ = 295 nm) of the compounds were subtracted from the corresponding overall spectra. The potential influence of the inner-filter effect on the measurements was evaluated with Equation (S6) [88] and was found negligible to affect the measurements.

The Stern–Volmer constant (K_SV_), the SA-quenching constant (K_q_) and the SA-binding constant (K) of the compounds were determined using the Stern–Volmer and Scatchard equations (Equations (S2), (S3) and (S7)) and corresponding plots (Figures S15–S18). In these calculations, the fluorescence lifetime of tryptophan in SAs (τ_ο_) was assumed to be 10^−8^ s [81]. All compounds displayed K_q_ values higher than 10^10^ M^−1^s^−1^ (by two to three orders of magnitude) (Table 6) consistent with a static quenching mechanism [81], which leads to the conclusion that compounds interact with the albumins.

The values of SA-binding constants for compounds were found to be in the order of 10^5^ M^−1^ (Table 6) consistent with values reported for a series of Co(II) compounds with fenamato ligands [27,28,29,32,33]. Among the complexes studied herein, complex **4** shows the highest binding constants for both albumins. The relatively high K values do not exceed the value of the association constants of avidin with a series of compounds (of the order 10^15^ M^−1^) which serve as a benchmark for reversible strong noncovalent interactions [89]. Thus, the SA-binding constants suggest reversible binding of the compounds to the albumins facilitating their transport of the complexes to biological targets and subsequent release.

#### 2.7.2. Determination of the Albumin-Binding Site

One of the primary functions of SAs is the transport of various compounds such as drugs, fatty acids and hormones. Albumins are composed of three domains, I, II and III, and each of them is subdivided into A and B subdomains that host compounds [90]. The most significant drug binding sites are Sudlow’s site I and Sudlow’s site II, located in subdomains IIA and IIIA, respectively [91]. Site markers like warfarin and ibuprofen are used to identify in which albumin site a compound is bound, as these markers bind selectively to Sudlow’s sites I and II, respectively. The SA-binding site of the compounds was investigated by calculating the SA-binding constant for both HSA and BSA in the presence of ibuprofen or warfarin and comparing them with the values previously derived (in the absence of any marker). The decrease in K value in the presence of a site marker indicates that the compound binds to the albumin competing with the corresponding marker for the same binding site [29,68,92].

Incremental addition of the compounds into a solution containing the SA (HSA or BSA) and the respective site marker (warfarin or ibuprofen) induced quenching of the initial fluorescence emission band (Figures S19–S22). The SA-binding constants for each compound in the presence of warfarin or ibuprofen were calculated with the Scatchard equation (Equation (S7)) and the corresponding plots (Figures S23–S26) and are compared with those calculated in the absence of any site marker to determine the preferable drug site.

In the case of HSA, a decrease in K values (Table 6) is observed for all the compounds in the presence of ibuprofen suggesting their preference to binding site II. Regarding BSA, only complex **2** shows a decreased binding constant in the presence of ibuprofen suggesting a selective binding to Sudlow’s site II, while 4′–NO_2_–fenH and complexes **1**, **3** and **4** seem to bind preferentially to Sudlow’s site I.

## 3. Experimental

### 3.1. Materials—Instrumentation–Physical Measurements

The reagents used in the current research were used as purchased from commercial sources: CoCl_2_·6H_2_O, phen, neoc, bipyam, BSA, HSA, EB, ABTS, K_2_CO_3_, CuSO_4_ and KOH from Sigma-Aldrich Co. (Burlington, MA, USA); 2–chlorobenzoic acid, and 4–nitroaniline from Alfa Aesar (Heysham, UK); CT DNA, DPPH, BHT, NDGA, K_2_S_2_O_8_ and trolox from J&K Scientific Co. (Beijing, China); sodium warfarin, ibuprofen and DPPH from TCI; NaCl and trisodium citrate from Merck (Rahway, NJ, USA); H_2_O_2_ (30% *w*/*v*) from PanReac AppliChem ITW Reagents Co. (Barcelona, Spain); supercoiled circular pBR322 plasmid DNA from New England Bioline (Ipswich, MA, USA); L-ascorbic acid, Na_2_HPO_4_, NaH_2_PO_4_, HCl (35% *v*/*v*) and solvents from Chemlab Co (Miami, FL, USA). All these chemicals and solvents were reagent grade.

CT DNA was diluted to buffer (15 mM trisodium citrate and 150 mM NaCl at pH 7.0) and was stirred to form the CT DNA stock solution. The solution of CT DNA gave a ratio of UV absorbance at 260 and 280 nm (A_260_/A_280_) of 1.87, and its concentration was determined from its absorbance at 260 nm (ε = 6600 M^−1^cm^−1^) [93] following a 1:20 dilution. The resultant stock solution was stored at 4 °C for no longer than ten days.

Fourier transform infrared (FT–IR) spectra were obtained using a Thermo Scientific (Waltham, MA, USA) Nicolet iS20 FT–IR ATR spectrometer in the range 400–4000 cm^−1^. The following abbreviations were used to denote peak intensities: vs = very strong; s = strong; m = medium; Δ*v*(COO) = *v*_asym_(COO) − *v*_sym_(COO). The UV-vis spectra were recorded in the range 200–800 nm as nujol mulls and in solution (concentrations in the range of 1 µM–5 mM) on a Jasco V–750 spectrophotometer equipped with internal thermostat. C, H and N elemental analysis were performed on a Perkin Elmer (Waltham, MA, USA) 240B elemental analyzer. ^1^H NMR spectra of 4′–NO_2_–fenH were recorded on an Agilent (Santa Clara, CA, USA) DD2 500 MHz spectrometer in DMSO–*d_6_* as solvent. The molar conductivity measurements of a DMSO solution (1 mM) of the complexes were performed on a Crison Basic (Alella, Spain) 30 conductometer. Fluorescence spectra of the compounds were recorded in solution on a Hitachi F–7000 fluorescence spectrophotometer. Viscosity experiments were carried out using an ALPHA L Fungilab rotational viscometer equipped with an 18 mL LCP spindle at 100 rpm.

### 3.2. Synthesis of the Compounds

#### 3.2.1. Synthesis of 4′–NO_2_–fenH

4′-NO_2_-fenH was prepared following modified procedures previously reported [34,35]. More specifically, 2–chlorobenzoic acid (3.6 mmol, 564 mg), 4–nitroaniline (7.2 mmol, 1000 mg), K_2_CO_3_ (1.8 mmol, 249 mg) and CuSO_4_ (20 mg, catalytic) were dissolved in DMF (6 mL) and refluxed at 153 °C for 24 h. The reaction mixture was allowed to cool to room temperature (RT) and 15 mL of 1 M HCl were added. A precipitate was formed and was collected after standing for 24 h at RT. The filtrate was washed with 200 mL H_2_O and was left to dry under vacuum. Green–brown solid, m.p. 121–124 °C (lit.: 120–123 °C [94]). ^1^H-NMR spectra are in correlation with reference [36] and recrystallized in EtOH; (86%) was collected. IR (ATR), *v*/cm^−1^: *ν*(O-H): 3301 (m), v(C=O)_carboxylic_ 1671 (s); δ(N–H): 1629 (m); ν(C-O)_carboxylic_ 1240 (s). ^1^H NMR (500 MHz, DMSO-*d*_6_), *δ* (ppm) (Figure S1): 13.33 (brs, 1H), 9.84 (s, 1H), 8.13 (d, *J* = 9.2 Hz, 2H), 7.96 (dd, *J* = 7.9, 1.7 Hz, 1H), 7.60–7.50 (m, 2H), 7.26 (d, *J* = 9.2 Hz, 2H), 7.11 (td, *J* = 8.1, 1.5 Hz, 1H).

#### 3.2.2. Synthesis of Complex [Co(4′–NO_2_–fen)_2_(MeOH)_4_] (Complex **1**)

A methanolic solution (12 mL) containing 4′–NO_2_–fenamic acid (0.3 mmol, 77 mg) and KOH (0.3 mmol, 0.3 mL, 1 M) was stirred for 1 h. The reaction solution was added to a methanolic solution (6 mL) of CoCl_2_∙6H_2_O (0.15 mmol, 35 mg). The resulting solution was stirred for 30 min and was allowed to slowly evaporate at room temperature. An orange (micro)crystalline product (yield: 65 mg, 70%) was formed and collected after a few days. Anal. Calc for [Co(4′–NO_2_–fen)_2_(MeOH)_4_] (C_30_H_34_CoN_4_O_12_) (MW = 701.55): C, 51.36; H, 4.89; N, 7.99%. Found: C, 51.65; H, 5.01; N, 7.84%. IR (ATR), *v*/cm^−1^: *v*_asym_(COO): 1584 (vs); *v*_sym_(COO): 1395 (m); Δ*v*(COO) = 189. UV-vis in DMSO solution, λ_max_/nm (ε/Μ^−1^cm^−1^): 670 (120), 391 (5400), 269 (12,500). The complex is soluble in MeOH, EtOH, DMF and DMSO (Λ_Μ_ = 18 S∙cm^2^∙mol^−1^ in 1 mM DMSO), and partially soluble in H_2_O and MeCN.

#### 3.2.3. Synthesis of Complexes **2**–**4**

Complexes **2**–**4** were synthesized in a similar way. More specifically, a solution containing 4′–NO_2_–fenamic acid (0.3 mmol, 77 mg) and KOH (0.3 mmol, 0.3 mL of 1 M solution in MeOH) was stirred for 1 h. Afterwards, the solution was added to a solution (3 mL) of CoCl_2_∙6H_2_O (0.15 mmol, 35 mg) simultaneously with a solution (3 mL) of the corresponding *N*,*N*′-donor (0.15 mmol) and the reaction solution was stirred for additional 30 min. After filtration, the solution was left to evaporate slowly at room temperature.

[Co(4′–NO_2_–fen)_2_(bipyam)] (complex **2**): The reaction took place in methanol; bipyam (0.15 mmol, 25 mg) was used as the corresponding *N*,*N*′-donor. An orange-colored (micro)crystalline product (yield: 50 mg, 45%) was collected after a week. Anal. Calc for [Co(4′–NO_2_–fen)_2_(bipyam)] (C_36_H_27_CoN_7_O_8_) (MW = 744.59): C, 58.07; H, 3.66; N, 13.17%. Found: C, 57.95; H, 3.91; N, 12.84%. IR (ATR), *v*/cm^−1^: *v*_asym_(COO): 1585 (vs); *v*_sym_(COO): 1395 (s); Δ*v*(COO) = 190; ρ(C–H)_bipyam_: 770 (m). UV-vis in DMSO solution, λ_max_/nm (ε/Μ^−1^cm^−1^): 480 (430), 397 (4200), 313 (6300), 270 (13,700). The complex is soluble in MeOH, EtOH, DMF and DMSO (Λ_Μ_ = 11 S∙cm^2^∙mol^−1^ in 1 mM DMSO), and partially soluble in H_2_O and MeCN.

[Co(4′–NO_2_–fen)_2_(phen)] (complex **3**): The reaction took place in a 1:1 methanol–dichloromethane mixture; phen (0.15 mmol, 27 mg) was used as the corresponding *N*,*N*′-donor. Brown (micro)crystalline product (yield: 62 mg, 55%) was formed after two weeks. Anal. Calc for [Co(4′–NO_2_–fen)_2_(phen)] (C_38_H_26_CoN_6_O_8_) (MW = 753.59): C, 60.57; H, 3.48; N, 11.15%. Found: C, 60.90; H, 3.31; N, 10.80%. IR (ATR), *v*/cm^−1^: *v*_asym_(COO): 1584 (s); *v*_sym_(COO): 1394 (s); Δ*v*(COO) = 190; ρ(C–H)_phen_: 725 (m). UV-vis in DMSO solution, λ_max_/nm (ε/Μ^−1^cm^−1^): 485 (280), 394 (3900), 272 (19,000). The complex is soluble in MeOH, EtOH, DMF and DMSO (Λ_Μ_ = 16 S∙cm^2^∙mol^−1^ in 1 mM DMSO), and partially soluble in H_2_O and MeCN.

[Co_2_(4′–NO_2_–fen)_2_(μ–CH_3_O)_2_(neoc)_2_] (complex **4**): The reaction took place in a 1:1 methanol–dichloromethane mixture; neoc (0.15 mmol, 31 mg) was used as the corresponding *N*,*N*′-donor. Brown crystals (yield: 30 mg, 35%) of the complex suitable for X-ray structure determination were formed and collected after a few days. Anal. Calc for [Co_2_(4′–NO_2_–fen)_2_(μ–CH_3_O)_2_(neoc)_2_] (C_56_H_48_Co_2_N_8_O_10_) (MW = 1110.91): C, 60.55; H, 4.36; N, 10.09%. Found: C, 60.75; H, 4.51; N, 9.88%. IR (ATR), *v*/cm^−1^: *v*_asym_(COO): 1593 (vs); *v*_sym_(COO): 1361 (s); Δ*v*(COO) = 232; ρ(C–H)_neoc_: 733 (m). UV-vis in DMSO solution, λ_max_/nm (ε/Μ^−1^cm^−1^): 495 (250), 427 (4200), 271 (18,700). The complex is soluble in MeOH, EtOH, DMF and DMSO (Λ_Μ_ = 9 S∙cm^2^∙mol^−1^ in 1 mM DMSO), and partially soluble in H_2_O and MeCN.

### 3.3. X-Ray Crystal Structure Determination

Single crystals of complex **4** suitable for analysis of the crystal structure were mounted at RT on a Bruker Kappa APEX2 diffractometer equipped with a Triumph monochromator using Mo Kα (λ = 0.71073 Å, source operating at 50 kV and 30 mA) radiation. Unit cell dimensions were determined and refined by using the angular settings of 183 high intensity reflections (>10σ(I)) in the range 10° < 2θ < 20°. Intensity data were recorded using φ and ω–scans. The crystal presented no decay during the data collection. The frames collected were integrated with the Bruker SAINT Software package (v 2) [95] using a narrow-frame algorithm. Data were corrected for absorption using the numerical method (SADABS) based on crystal dimensions [96]. The structure was solved using SUPERFLIP [97] incorporated in Crystals. Data refinement (full-matrix least-squares methods on *F*^2^) and all subsequent calculations were carried out using the Crystals version 14.61 build 6720 program package [98]. All non-hydrogen atoms were refined anisotropically. Hydrogen atoms were located from difference Fourier maps and refined using soft constraints at idealized positions riding on the parent atoms with isotropic displacement parameters U_iso_(H) = 1.2U_eq_(C) and 1.5U_eq_ (–NH hydrogens) at distances C–H 0.95 Å and N–H 0.86 Å. NH hydrogen atoms were free to rotate. Crystallographic data for the complex are presented in Table S1.

Further details on the crystallographic studies as well as atomic displacement parameters are given as Supporting Information in the form of cif file. CCDC deposition number 2452302 contains the supplementary crystallographic data for complex **4**. These data can be obtained free of charge via www.ccdc.cam.ac.uk (accessed on 13 June 2025) (or from the Cambridge Crystallographic Data Centre, 12 Union Road, Cambridge CB21EZ, UK; fax: (+44) 1223–336–033; or deposit@ccdc.cam.ac.uk).

### 3.4. In Vitro Biological Studies of the Complexes

The in vitro biological activity (i.e., antioxidant and antimicrobial activity, interaction with CT DNA, pDNA and albumins) of the compounds (4′–NO_2_–fenH and complexes **1**–**4**) was studied by dissolving them in DMSO (1 mM). The use of DMSO was necessary due to the low aqueous solubility of the compounds. The mixing of such solutions with the aqueous buffer solutions containing DNA or albumins never exceeded 5% DMSO (*v*/*v*) in the final solution. Control experiments were carried out to assess the impact of the DMSO on the measurements and no significant effect was observed.

All the procedures and relevant equations used in the in vitro study of the biological activity (antioxidant activity, antimicrobial activity and interaction with CT DNA, plasmid DNA, HSA and BSA) of the compounds can be found in the Supporting Information file (Sections S1–S5).

## 4. Conclusions

A nitro-derivative of fenamic acid (4′-NO_2_-fenH) and a series of Co(II) complexes in the presence of the *N*,*N*′-donors 1,10–phenanthroline, 2,9–dimethyl–1,10–phenanthroline and 2,2′–dipyridylamine as co-ligands have been isolated and characterized with a series of spectroscopic techniques including IR, UV-vis and ^1^H NMR spectroscopies. The crystal structure of the dinuclear complex [Co_2_(4′–NO_2_–fen)_2_(μ–CH_3_O)_2_(neoc)_2_] (complex **4**) was determined with single-crystal X-ray crystallography. Complex **4** is among the dinuclear Co(II) complexes containing two methoxo bridges found in the literature.

The compounds were evaluated in vitro for their antioxidant and antimicrobial potency as well as their interaction with biomacromolecules such as DNA and albumins. Regarding the antioxidant activity of the compounds, the compounds are inactive towards DPPH radicals, showed moderate scavenging activity of the ABTS radicals (ABTS% = 21.39–48.12%) and were found more active towards H_2_O_2_ (H_2_O_2_% = 66.46–77.59%) than the reference compound L-ascorbic acid. Regarding the antimicrobial potency of the compounds, their activity was examined against four bacteria strains (*X. campestris*, *E. coli*, *B. subtilis* and *S. aureus*). According to the MIC values, the complexes presented similar activity as 4′-NO_2_-fenH did against all bacterial strains tested. The best activity was found for complex **4** against most bacterial strains tested (MIC value of 11 μM).

The complexes may interact with CT DNA via intercalation (as concluded from the techniques employed) and tightly showing K_b_ values in the range 7.20 × 10^4^–2.06 × 10^6^ M^−1^ magnitude with the highest DNA-binding constant found for complex **4** (K_b_ = 2.06 × 10^6^ M^−1^). The ability of the compounds to cleave pBR322 plasmid DNA to relaxed circular DNA is moderate at the concentration of 500 μM, and is significantly enhanced upon irradiation with UVB, UVA or visible light. Complex **3** is an active pDNA-photocleaver upon irradiation with the lights used herein. The binding of the compounds with both albumins tested is reversible but tight, showing their ability to get transported and released at potential biotargets. Competitive studies with the typical site markers warfarin and ibuprofen showed that, in the case of HSA, all compounds seem to bind preferentially to Sudlow’s site II. In the case of BSA, only complex **2** keeps the preference for binding site II while 4′–NO_2_–fenH and complexes **1**, **3** and **4** seem to alternate their binding selectivity towards Sudlow’s site I.

In total, the introduction on the nitro group on fenamic acid did not yield a more active antioxidant agent than the commercial fenamate NSAIDs Hmef, Htolf, Hfluf and Hmeclf. On the other hand, the Co(II) complexes of the NO_2_–fenamic derivative were more active than free 4′–NO_2_–fenH, and in the range found for the Co(II) analogues of the commercial fenamates. Regarding the interaction with CT DNA, the nitro-derivative was proved to be a tighter DNA-binder (with higher K_b_ value) than the commercial fenamates while the interaction with the albumins revealed similar albumin-binding strength (K values of the same magnitude). Furthermore, the DNA- and SA-binding constants of complexes **1**–**4** were found to be of the same magnitude with their mefenamato, tolfenamato, meclofenamato and flufenamato Co(II)-analogues.

These results may reveal promising properties of the synthesized compounds, adding them to the arsenal of candidate compounds for possible applications as antioxidants, drug carriers or anticancer drugs. In such a case, the in vivo efficacy of the compounds should be further evaluated with more elaborate studies.

## Figures and Tables

**Table 1:** Selected bond lengths (Å) and angles (°) for complex **4**.

Bonds	Length (Å)	Bonds	Length (Å)
Co1—O1	2.0008 (17)	Co1—N1	2.1155 (19)
Co1—O5	1.9733 (16)	Co1—N2	2.1533 (19)
Co1—O5^i^	2.0098 (16)	Co1…Co1^i^	3.122
**Bonds**	**Angle (°)**	**Bonds**	**Angle (°)**
O5^i^—Co1—N1	119.67 (7)	N1—Co1—N2	77.91 (8)
O5^i^—Co1—N2	161.19 (7)	N1—Co1—O1	109.13 (7)
O5^i^—Co1—O1	90.88 (7)	N1—Co1—O5	109.65 (7)
O5^i^—Co1—O5	76.78 (7)	N2—Co1—O1	89.06 (8)
O1—Co1—O5	140.41 (8)	N2—Co1—O5	91.41 (7)
Co1^i^—O5—Co1	103.22 (7)		

Symmetry code: (i) −*x* + 1, −*y* + 1, −*z* + 1.

**Table 2:** %DPPH-scavenging ability (DPPH%), %ABTS radical scavenging activity (ABTS%) and H_2_O_2_-reducing ability (H_2_O_2_%) for the compounds.

Compound	DPPH%, 30 min/60 min	ABTS%	H_2_O_2_%
4′–NO_2_–fenH	Not active	Not active	66.46 ± 0.33
Complex **1**	Not active	24.68 ± 0.35	72.68 ± 0.63
Complex **2**	Not active	43.65 ± 0.37	77.59 ± 0.28
Complex **3**	Not active	48.12 ± 0.88	69.48 ± 0.37
Complex **4**	Not active	21.39 ± 0.22	72.44 ± 0.42
NDGA	94.33 ± 0.11/90.47 ± 0.42	Not tested	Not tested
BHT	70.83 ± 0.03/83.39 ± 0.39	Not tested	Not tested
Trolox	Not tested	96.50 ± 0.05	Not tested
L-ascorbic acid	Not tested	Not tested	58.90 ± 0.52

**Table 3:** MIC values (in μg/mL and in μM (values in parentheses)) for 4′–NO_2_–fenH and its complexes **1**–**4**.

Compound	*X. campestris*	*E. coli*	*B. subtilis*	*S. aureus*
4′–NO_2_–fenH	25 (97)	25 (97)	12.5 (48)	25 (97)
Complex **1**	>50 (>71)	>50 (>71)	12.5 (18)	12.5 (18)
Complex **2**	25 (33)	>50 (>67)	25 (34)	>50 (>67)
Complex **3**	25 (33)	25 (33)	25 (33)	25 (33)
Complex **4**	12.5 (11)	>50 (>45)	12.5 (11)	12.5 (11)

**Table 4:** UV-vis spectroscopic data concerning the interaction of 4′–NO_2_–fenH and its complexes **1**–**4** with CT DNA: UV–band (λ_max_, nm) (percentage of the observed hyper-/hypochromism (ΔA/A_0_, %), blue-/red-shift in λ_max_ (Δλ, nm)); DNA-binding constant (K_b_, M^−1^); *T*_m_ (in °C).

Compound	λ_max_ (nm) (ΔA/A_0_ (%)) ^a^, Δλ (nm) ^b^)	K_b_ (M^−1^)	*T*_m_ (°C)
4′–NO_2_–fenH	400 (−12 ^a^, +20 ^b^)	1.62 (±0.09) × 10^6^	83.7
Complex **1**	391 (−10 ^a^, 0 ^b^)	7.20 (±0.15) × 10^4^	83.7
Complex **2**	321 (+4, −0.5); 396.5 (−10, 0)	7.72 (±0.04) × 10^4^	74.5
Complex **3**	393 (−2.5, +0.5)	1.29 (±0.05) × 10^5^	84.7
Complex **4**	421 (−3, −4.5)	2.06 (±0.41) × 10^6^	73.9

^a^ “+” denotes hyperchromism; “−” denotes hypochromism. ^b^ “+” denotes red-shift; “−” denotes blue-shift.

**Table 5:** Fluorescence features of the EB-displacement studies of 4′–NO_2_–fenH and its complexes **1**–**4**: percentage of EB–DNA fluorescence emission quenching (ΔI/I_0_, in %), Stern–Volmer (K_SV_, in M^−1^) and quenching constants (K_q_, in M^−1^s^−1^).

Compound	ΔI/I_0_ (%)	K_sv_ (M^−1^)	K_q_ (M^−1^s^−1^)
4′–NO_2_–fenH	47.1	5.36 (±0.27) × 10^4^	2.33 (±0.11) × 10^12^
Complex **1**	64.6	4.99 (±0.14) × 10^4^	2.17 (±0.06) × 10^12^
Complex **2**	67.3	8.91 (±0.21) × 10^4^	3.88 (±0.09) × 10^12^
Complex **3**	73.7	7.39 (±0.21) × 10^4^	3.21 (±0.09) × 10^12^
Complex **4**	74.0	7.78 (±0.24) × 10^4^	3.38 (±0.10) × 10^12^

**Table 6:** The BSA/HSA-quenching constants (K_q_, in M^−1^s^−1^) calculated for 4′–NO_2_–fenH and its complexes **1**–**4**. The BSA/HSA-binding constants (K, in M^−1^) calculated for 4′–NO_2_–fenH and its complexes **1**–**4** in the absence or presence of the albumin-site markers warfarin and ibuprofen.

Compound	K_q (BSA)_ (M^−1^s^−1^)	K_(BSA)_ (M^−1^)	K_(BSA, warfarin)_ (M^−1^)	K_(BSA, ibuprofen)_ (M^−1^)
4′–NO_2_–fenH	1.18 (±0.09) × 10^13^	3.31 (±0.14) × 10^5^	1.41 (±0.06) × 10^5^	2.70 (±0.11) × 10^5^
Complex **1**	9.51 (±0.06) × 10^12^	1.68 (±0.07) × 10^5^	1.06 (±0.04) × 10^5^	1.38 (±0.08) × 10^5^
Complex **2**	1.30 (±0.06) × 10^13^	1.75 (±0.08) × 10^5^	1.85 (±0.06) × 10^5^	1.42 (±0.09) × 10^5^
Complex **3**	1.64 (±0.04) × 10^13^	2.06 (±0.07) × 10^5^	1.60 (±0.06) × 10^5^	1.95 (±0.03) × 10^5^
Complex **4**	2.19 (±0.05) × 10^13^	2.74 (±0.10) × 10^5^	2.40 (±0.06) × 10^5^	3.60 (±0.14) × 10^5^
	**K_q (HSA)_ (M^−1^s^−1^)**	**K_(HSA)_ (M^−1^)**	**K_(HSA, warfarin)_ (M^−1^)**	**K_(HSA, ibuprofen)_ (M^−1^)**
4′–NO_2_–fenH	9.54 (±1.09) × 10^12^	3.82 (±0.09) × 10^5^	3.22 (±0.12) × 10^5^	2.91 (±0.06) × 10^5^
Complex **1**	1.10 (±0.03) × 10^13^	1.68 (±0.04) × 10^5^	1.99 (±0.11) × 10^5^	1.66 (±0.09) × 10^5^
Complex **2**	1.91 (±0.06) × 10^13^	2.56 (±0.10) × 10^5^	2.75 (±0.19) × 10^5^	2.02 (±0.03) × 10^5^
Complex **3**	1.87 (±0.04) × 10^13^	1.93 (±0.09) × 10^5^	4.80 (±0.17) × 10^5^	5.23 (±0.17) × 10^4^
Complex **4**	3.98 (±0.12) × 10^13^	5.03 (±0.19) × 10^5^	5.00 (±0.18) × 10^5^	3.04 (±0.15) × 10^5^

## Data Availability

All necessary supplementary data are included in the Supplementary Files.

## Supplementary Materials

Supplementary data associated with this article can be found in the online version, at https://www.mdpi.com/article/10.3390/molecules30122621/s1. Cif and checkcif files for compound **4**. Protocols and equations regarding antioxidant activity assay (S1), antibacterial activity studies (S2), binding studies with CT DNA (S3), pDNA cleavage studies (S4), and albumin-binding studies (S5), Table S1 and Figures S1–S26 are included in the ESI file [99,100,101,102,103,104,105].

## Abbreviations

The following abbreviations are used in this manuscript:

4′–NO_2_–fen^−^	4′–nitrofenamato anion
4′–NO_2_–fenH	4′–nitrofenamic acid
ABTS	2,2′–azinobis–(3–ethylbenzothiazoline–6–sulfonic acid)
*B. subtilis*	*Bacillus subtilis* ATCC 6633
BHT	butylated hydroxytoluene
bipyam	2,2′–bipyridylamine
BSA	bovine serum albumin
COX	cyclooxygenase
CT	calf-thymus
dicl^−^	anion of diclofenac
DPPH	1,1–diphenyl–picrylhydrazyl
Ds	double-stranded
*E. coli*	*Escherichia coli* XL1
EB	ethidium bromide
fen^−^	fenamato anion
fluf^−^	flufenamato anion
H_2_difl	diflunisal
Hdicl	diclofenac
Hdifl^−^	anion of diflunisal
Hfen	fenamic acid
Hfluf	flufenamic acid
Himi	1H–imidazole
Hmeclf	meclofenamic acid
Hmef	mefenamic acid
Hnap	naproxen
Hnif	niflumic acid
HSA	human serum albumin
Htolf	tolfenamic acid
K	SA-binding constant
K_b_	DNA-binding constant
K_q_	quenching constant
K_SV_	Stern–Volmer constant
meclf^−^	meclofenamato anion
mef^−^	mefenamato anion
MIC	minimum inhibitory concentration
nap^−^	anion of naproxen
NDGA	nordihydroguaiaretic acid
neoc	2,9–dimethyl–1,10–phenanthroline (neocuproine)
nif^−^	niflumato anion
NSAID	non–steroidal anti-inflammatory drug
pDNA	pBR322 plasmid DNA
phen	1,10–phenanthroline
*S. aureus*	*Staphylococcus aureus* ATCC 29213
SA	serum albumin
ss	single-stranded
*T*m	DNA-melting temperature
tolf^−^	tolfenamato anion
trolox	6–hydroxy–2,5,7,8–tetramethylchroman–2–carboxylic acid
*X. campestris*	*Xanthomonas campestris* ATCC 33013
Δν(COO)	νasym(COO) − νsym(COO)
