# Supplementary material for: Cobalt(II) Complexes of 4′–Nitro–Fenamic Acid: Characterization and Biological Evaluation
Source: Molecules. 2025 Jun 17;30(12):2621. doi: 10.3390/molecules30122621 (PMC12195967; doi:10.3390/molecules30122621)

checkCIF/PLATON report

Structure factors have been supplied for datablock(s) I

THIS REPORT IS FOR GUIDANCE ONLY. IF USED AS PART OF A REVIEW PROCEDURE FOR PUBLICATION, IT SHOULD NOT REPLACE THE EXPERTISE OF AN EXPERIENCED CRYSTALLOGRAPHIC REFEREE.

No syntax errors found. CIF dictionary Interpreting this report

Datablock: I

Bond precision: C-C = 0.0043 A Wavelength=0.71073

Cell:	a=8.3973(17)	b=12.647(3)	c=13.317(3)
	alpha=68.615(8)	beta=72.649(8)	gamma=74.349(8)
Temperature:	295 K		

	Calculated	Reported
Volume	1236.2(5)	1236.1(4)
Space group	P -1	P -1
Hall group	-P 1	?
Moiety formula	C56 H48 Co2 N8 O10	C56 H48 Co2 N8 O10
Sum formula	C56 H48 Co2 N8 O10	C56 H48 Co2 N8 O10
Mr	1110.88	1110.91
Dx,g cm-3	1.492	1.492
Z	1	1
Mu (mm-1)	0.742	0.742
F000	574.0	574.0
F000'	574.92	
h,k,lmax	10,15,16	10,15,16
Nref	4962	4840
Tmin,Tmax	0.907,0.956	0.920,0.960
Tmin'	0.837	

Correction method= # Reported T Limits: Tmin=0.920 Tmax=0.960
AbsCorr = NUMERICAL

Data completeness= 0.975 Theta(max)= 26.167

R(reflections)= 0.0350(4074) wR2(reflections)= 0.0650(4074)

S = 1.002 Npar= 343

The following ALERTS were generated. Each ALERT has the format
test-name_ALERT_alert-type_alert-level.
Click on the hyperlinks for more details of the test.

Alert level C

```
PLAT220_ALERT_2_C NonSolvent   Resd 1  O   Ueq(max)/Ueq(min) Range        3.2 Ratio
PLAT910_ALERT_3_C Missing # of FCF Reflection(s) Below Theta(Min).         10 Note
                1  0  0,   0  1  0,   1  1  0,   0 -1  1,   0  0  1,   1  0  1,
                0  1  1,   1  1  1,   0  0  2,   0  1  2,
PLAT911_ALERT_3_C Missing FCF Refl Between Thmin & STh/L=    0.600          8 Report
               -7  7  0,  -1 -3  1,  -1 -1  1,   0  2  1,  -7  7  1,  -6  8  1,
               -6  9  1,   1  1  2,
```

Alert level G

```
PLAT007_ALERT_5_G Number of Unrefined Donor-H Atoms .............           1 Report
              H31
PLAT154_ALERT_1_G The s.u.'s on the Cell Angles are Equal ..(Note)      0.008 Degree
PLAT769_ALERT_4_G CIF Embedded Explicitly Supplied Scattering Data     Please Note
PLAT794_ALERT_5_G Tentative Bond Valency for Co1       (II)      .       1.98 Info
PLAT808_ALERT_5_G No Parseable SHELXL Style Weighting Scheme Found     Please Check
PLAT912_ALERT_4_G Missing # of FCF Reflections Above STh/L=  0.600        114 Note
PLAT929_ALERT_5_G No Weight Pars,Obs and Calc R1,wR2,S not Checked          ! Info
PLAT960_ALERT_3_G Number of Intensities with I < - 2*Sigma(I) ....         64 Check
PLAT969_ALERT_5_G The 'Henn et al.' R-Factor-gap value ..........       2.450 Note
              Predicted wR2: Based on SigI**2  2.65 or SHELX Weight  7.11
```

0 **ALERT level A** = Most likely a serious problem - resolve or explain
0 **ALERT level B** = A potentially serious problem, consider carefully
3 **ALERT level C** = Check. Ensure it is not caused by an omission or oversight
9 **ALERT level G** = General information/check it is not something unexpected

1 ALERT type 1 CIF construction/syntax error, inconsistent or missing data
1 ALERT type 2 Indicator that the structure model may be wrong or deficient
3 ALERT type 3 Indicator that the structure quality may be low
2 ALERT type 4 Improvement, methodology, query or suggestion
5 ALERT type 5 Informative message, check

It is advisable to attempt to resolve as many as possible of the alerts in all categories. Often the minor alerts point to easily fixed oversights, errors and omissions in your CIF or refinement strategy, so attention to these fine details can be worthwhile. In order to resolve some of the more serious problems it may be necessary to carry out additional measurements or structure refinements. However, the purpose of your study may justify the reported deviations and the more serious of these should normally be commented upon in the discussion or experimental section of a paper or in the "special_details" fields of the CIF. checkCIF was carefully designed to identify outliers and unusual parameters, but every test has its limitations and alerts that are not important in a particular case may appear. Conversely, the absence of alerts does not guarantee there are no aspects of the results needing attention. It is up to the individual to critically assess their own results and, if necessary, seek expert advice.

Publication of your CIF in IUCr journals

A basic structural check has been run on your CIF. These basic checks will be run on all CIFs submitted for publication in IUCr journals (*Acta Crystallographica*, *Journal of Applied Crystallography*, *Journal of Synchrotron Radiation*); however, if you intend to submit to *Acta Crystallographica Section C* or *E* or *IUCrData*, you should make sure that full publication checks are run on the final version of your CIF prior to submission.

Publication of your CIF in other journals

Please refer to the *Notes for Authors* of the relevant journal for any special instructions relating to CIF submission.

PLATON version of 02/02/2025; check.def file version of 02/02/2025

Datablock I - ellipsoid plot

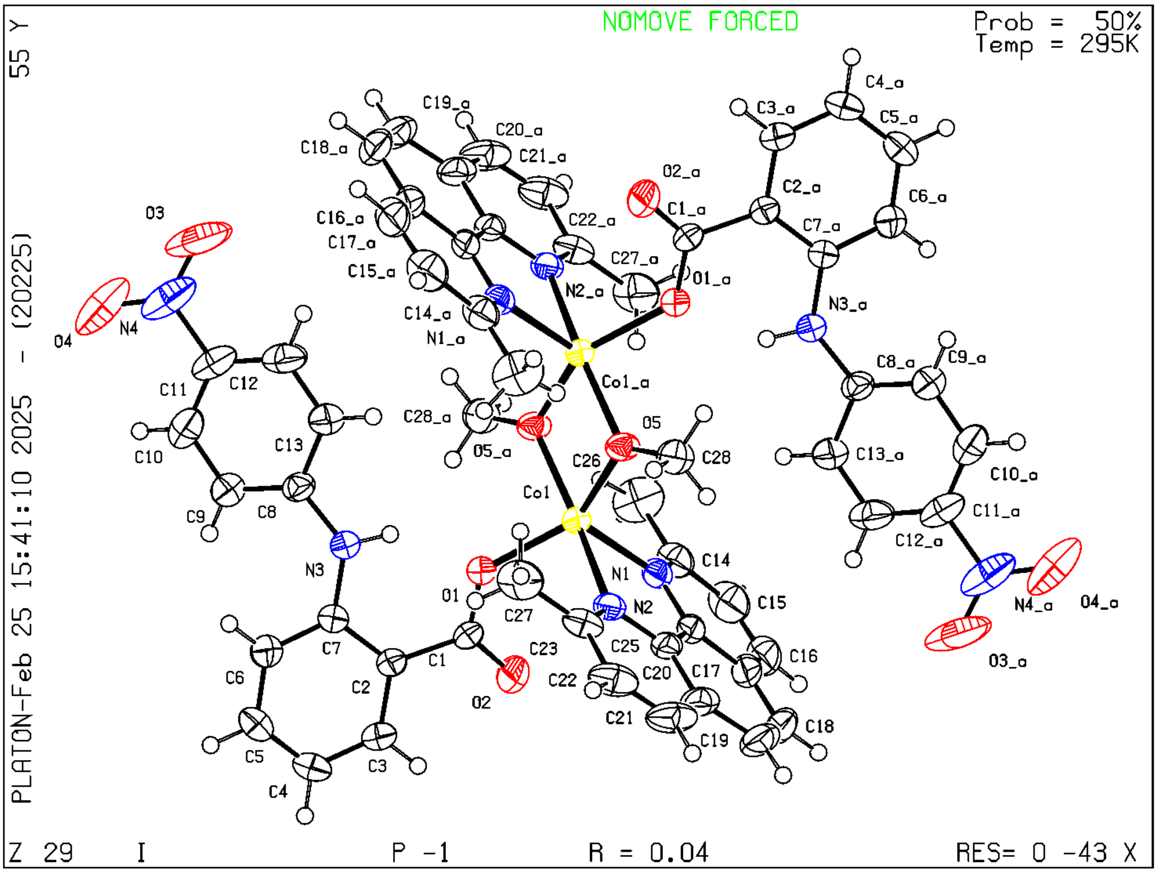

Supplement: Supplementary file 1 [file molecules-30-02621-s001.zip › molecules-3700019-supplementary/molecules-3700019, Checkcif of complex 4.pdf]
