# Supplementary material for: Cobalt(II) Complexes of 4′–Nitro–Fenamic Acid: Characterization and Biological Evaluation
Source: Molecules. 2025 Jun 17;30(12):2621. doi: 10.3390/molecules30122621 (PMC12195967; doi:10.3390/molecules30122621)
Supplement: Supplementary file 1 [file molecules-30-02621-s001.zip › molecules-3700019-supplementary/molecules-3700019, ESI.pdf]

Cobalt(II) Complexes of 4′–Nitro–Fenamic Acid: Characteriza-tion and Biological Evaluation

Georgios Malis 1, Antigoni Roussa 1, Efstathia Aikaterini Papantopoulou 2, Stavros Kalogiannis 2, Antonios G. Hatzidimitriou 1, Konstantina C. Fylaktakidou 3,* and George Psomas 1,*

1 Laboratory of Inorganic Chemistry, Department of Chemistry, Aristotle University of Thessaloniki, 54124 Thessaloniki, Greece

2 Department of Nutritional Sciences and Dietetics, International Hellenic University, Sindos, 57400 Thessalo-nikis, Greece

3 Laboratory of Organic Chemistry, Department of Chemistry, Aristotle University of Thessaloniki, 54124 Thessaloniki, Greece

*Correspondence: kfylakta@chem.auth.gr (K.C.F.); gepsomas@chem.auth.gr (G.P.)

SUPPLEMENTARY MATERIAL

Content

EXPERIMENTAL PROTOCOLS 4

S1 Antioxidant activity assay 4

S1.1 Determination of the reducing activity of the radical DPPH 4

S1.2 Assay of radical cation ABTS−scavenging activity 4

S1.3 Reduction of hydrogen peroxide 4

S2 Antibacterial Activity studies 5

S3 Binding studies with CT DNA 6

S3.1 Binding study with CT DNA by UV−vis spectroscopy 6

S3.2 Determination of the DNA−melting temperature (T_m) 6

S3.3 CT DNA−binding studies by viscosity measurements 6

S3.4 EB−displacement studies 7

S4 Plasmid DNA cleavage experiments 7

S5 Albumin−binding studies 8

S5.1 Interaction with albumins 8

S5.2 Competitive albumin−fluorescence studies with warfarin and ibuprofen 9

S6 References 10

TABLES 11

Table S1. Experimental crystallographic details for complex **4**. 11

FIGURES 12

Figure S1. ^{1}H NMR spectrum of 4′–NO_2–fenH in DMSO-d_6. 12

Figure S2. IR spectra (ATR) of the compounds. 13

Figure S3: Proposed structures for complexes **1–3**. 15

Figure S4. UV−vis spectra of a DMSO solution of the compounds in the presence of increasing amounts of CT DNA. 16

Figure S5. Plots of [DNA]$(\varepsilon A - \varepsilon f)$ *versus* [DNA] for the compounds. 17

Figure S6. Thermal melting profile of CT DNA in the absence or presence of the compounds. 18

Figure S7. Fluorescence emission spectra for EB–DNA in buffer solution in the absence and presence of increasing amounts of the compounds. 19

Figure S8. Stern−Volmer plots of the EB−DNA quenching experiments upon addition of the compounds. 20

Figure S9: Agarose gel electrophoretic pattern of EB–stained plasmid DNA (pBR322 plasmid DNA) after incubation with the compounds, in dark. 21

Figure S10: Agarose gel electrophoretic pattern of EB–stained plasmid DNA (pBR322 plasmid DNA) with the compounds, after irradiation at 312 nm (UV–B) for 30 min.......22

Figure S11: Agarose gel electrophoretic pattern of EB–stained plasmid DNA (pBR322 plasmid DNA) with the compounds, after irradiation at 365 nm (UV–A) for 30 min. 23

Figure S12: Agarose gel electrophoretic pattern of EB–stained plasmid DNA (pBR322 plasmid DNA) with the compounds, after irradiation under visible light for 2 h. 24

Figure S13. Fluorescence emission spectra of BSA in buffer solution in the presence of increasing amounts of the compounds. 25

Figure S14. Fluorescence emission spectra of HSA in buffer solution in the presence of increasing amounts of the compounds. 26

Figure S15. Stern−Volmer plots of the BSA–quenching experiments upon addition of the compounds...27

Figure S16. Stern−Volmer plots of the HSA–quenching experiments upon addition of the compounds...28

Figure S17. Scatchard plots of the BSA–quenching experiments upon addition of the compounds...29

Figure S18. Scatchard plots of the HSA–quenching experiments upon addition of the compounds...30

Figure S19. Fluorescence emission spectra of BSA in buffer solution in the presence of ibuprofen upon addition of increasing amounts of the compounds..................................31

Figure S20. Fluorescence emission spectra of BSA in buffer solution in the presence of warfarin upon addition of increasing amounts of the compounds. 32

Figure S21. Fluorescence emission spectra of HSA in buffer solution in the presence of ibuprofen upon addition of increasing amounts of the compounds..................................33

Figure S22. Fluorescence emission spectra of HSA in buffer solution in the presence of warfarin upon addition of increasing amounts of the compounds. 34

Figure S23. Scatchard plots of the BSA quenching experiments in the presence of ibuprofen upon addition of the compounds. 35

Figure S24. Scatchard plots of the BSA quenching experiments in the presence of warfarin upon addition of the compounds. 36

Figure S25. Scatchard plots of the HSA quenching experiments in the presence of ibuprofen upon addition of the compounds. 37

Figure S26. Scatchard plots of the HSA quenching experiments in the presence of warfarin upon addition of the compounds. 38

EXPERIMENTAL PROTOCOLS

S1 Antioxidant activity assay

The antioxidant activity of the compound was evaluated *via* the ability to scavenge *in vitro* free radicals such as DPPH and ABTS and to reduce H_2O_2. All the experiments were carried out at least in triplicate and the standard deviation of absorbance was less than 10% of the mean.

S1.1 Determination of the reducing activity of the radical DPPH

To an ethanolic solution of DPPH (0.1 mM) an equal volume solution of the compounds (0.1 mM) in ethanol was added. Absolute ethanol was also used as control solution. The absorbance at 517 nm was recorded at room temperature after 30 and 60 min in order to examine the possible existence of a potential time–dependence of the DPPH radical scavenging activity.[1] The DPPH–scavenging activity of the compounds was expressed as the percentage reduction of the absorbance values of the initial DPPH solution (DPPH%). NDGA and BHT were used as reference compounds.

S1.2 Assay of radical cation ABTS–scavenging activity

The ABTS assay was performed to determine the activity of the compounds to scavenge the radical cation ABTS. Initially, a water solution of ABTS was prepared (2 mM). ABTS radical cation ($ABTS^{+\cdot}$) was produced by the reaction of ABTS stock solution with potassium persulfate (0.17 mM) and the mixture was stored in the dark at room temperature for 12–16 h before its use. The ABTS was oxidized incompletely because the stoichiometric reaction ratio of ABTS and potassium persulfate is 1:0.5. The absorbance became maximal and stable only after more than 6 h of reaction although the oxidation of the ABTS started immediately. The radical was stable in this form for more than 2 days when allowed to stand in the dark at room temperature. Afterwards, the $ABTS^{+\cdot}$ solution was diluted in ethanol to an absorbance of 0.70 at 734 nm and 10 μL of diluted compounds or standards (0.1 mM) in DMSO were added. The absorbance was recorded out exactly 1 min after initial mixing [1]. The ABTS–radical scavenging activity of the compounds was expressed as the percentage inhibition of the absorbance of the initial ABTS solution (ABTS%). Trolox was used as an appropriate standard.

S1.3 Reduction of hydrogen peroxide

The ability of the compounds to reduce hydrogen peroxide (H_2O_2) was estimated according to the method described in the literature [2]. The reaction mixture contained 20 μL of each of the tested compounds (0.1 mM) and 5 μL H_2O_2 solution (40 mM) in phosphate

buffer (50 mM, pH 7.4). The absorbance was measured at 230 nm after 10 min. The antioxidant activity (reduction of H_2O_2) of the compounds was expressed as the percentage decrease of the initial H_2O_2 solution (H_2O_2%). L−ascorbic acid (or vitamin C) was used as a standard.

S2 Antibacterial Activity studies

The antibacterial activity of the compounds was evaluated by determining their respective MIC values towards two Gram–(–) (*Escherichia coli XL1* (*E. coli*) and *Xanthomonas campestris* ATCC 1395 (*X. campestris*)) and two Gram–(+) (*Staphylococcus aureus ATCC 29213* (*S. aureus*) and *Bacillus subtilis ATCC 6633* (*B. subtilis*)) bacterial species. Cultures of these microbial strains were grown on a rich selective agar medium and stored at 4°C. The selective media used were Nutrient Agar or Broth for *B. subtilis* and *S. aureus*, Yeast Mold Agar or Broth for *X. campestris* and Luria Agar or Broth for *E. coli*. Cells picked from the surface of the stored cultures were used to initiate liquid pre–cultures of the same selective medium at an initial turbidity of roughly 1 McFarland unit.

Pre–cultures were incubated for 24 h in a rotary shaking incubator and subsequently they were used to inoculate the test cultures used for the determination of MIC at an initial turbidity of 0.5 McFarland units. The test cultures consisted of Mueller–Hinton broth (Deben Diagnostics Ltd) containing different concentrations of the compounds. Different concentrations were achieved as follows: the compounds were freshly dissolved in DMSO to a concentration of 1 mg/mL, and they were diluted with DMSO, using the method of progressive double dilution. Therefore, working solutions with decreasing concentrations of the compounds under investigation were achieved.

The working solutions were subsequently diluted to the final desired concentration by addition to the growth medium at a proportion of 2:98. MIC values were determined as the lowest concentrations of the tested compounds that inhibited visible growth of each respective organism after a 24 h incubation [3]. Bacterial growth was determined by measuring the turbidity of appropriately diluted cultures at 600 nm with reference to equally diluted sterile growth medium and the inhibition achieved was calculated by comparing the turbidity of each culture to the average of the turbidity of three non–inhibited cultures. All test cultures were grown in triplicates and for the determination of MIC, growth had to be inhibited in at least two cultures of the triplicate. The incubation temperature at all stages was 37°C except for *X. campestris* that was cultivated at 28°C [4].

S3 Binding studies with CT DNA

In order to study the interaction of the compounds with DNA, the compound was initially dissolved in DMSO (1 mM). Mixing of such solutions with the aqueous buffer solutions of DNA used in the studies never exceeded 5% DMSO (v/v) in the final solution, which was needed due to low aqueous solubility of most compounds. In all experiments, the effect of DMSO on the data has been taken into consideration and the appropriate corrections have been performed. The interaction of the compound with CT DNA was monitored by UV−vis spectroscopy, and viscosity measurements, and *via* competitive studies with EB by fluorescence emission spectroscopy.

S3.1 Binding study with CT DNA by UV−vis spectroscopy

The interaction of the compound with CT DNA has been studied by UV−vis spectroscopy in order to investigate the possible binding mode to CT DNA and to calculate the DNA−binding constant (K_b). of the compounds K_b (in M^{-1}) was determined with the Wolfe−Shimer equation (equation S1) [5] and the plots [DNA]/(ε_A−ε_f) *versus* [DNA] using the UV−vis spectra of the compound (25–100 μM) recorded for a constant concentration with increasing amounts of CT DNA for diverse [compound]/[DNA] mixing ratios (= *r*). According to the Wolfe−Shimer equation:

$$\frac{[\mathrm{DNA}]}{(\varepsilon_A-\varepsilon_f)} = \frac{[\mathrm{DNA}]}{(\varepsilon_b-\varepsilon_f)} + \frac{1}{K_b(\varepsilon_b-\varepsilon_f)} \qquad \text{(equation S1)}$$

where [DNA] is the concentration of DNA in base pairs, ε_A = A_{obsd}/[compound], ε_f = the extinction coefficient for the free compound and ε_b = the extinction coefficient for the compound in the fully bound form. K_b is given by the ratio of slope to the y intercept in plots [DNA]/(ε_A−ε_f) *versus* [DNA].

S3.2 Determination of the DNA−melting temperature (T_m)

The UV−vis spectra of a CT DNA solution (1.25×10^{-4} M) in the presence of the compounds (2.5×10^{-6} M) were recorded upon increasing the temperature. The obtained data are presented as normalized absorbance at 260 nm (Normalized $A_{(260\ nm)}$) *versus* temperature (T (ºC)). A sigmoidal fitting of the data was applied for each compound and the melting temperature T_m (temperature with normalized absorbance of 0.5) was determined.

S3.3 CT DNA−binding studies by viscosity measurements

The interaction of compounds with DNA was evaluated *via* the study of the CT DNA viscosity ([DNA] = 0.1 mM) in a buffer solution (150 mM NaCl and 15 mM trisodium citrate at pH 7.0) in the presence of increasing amounts of the compounds (up to the value of *r* = 0.26). The obtained data are presented as $(\eta/\eta_0)^{1/3}$ *versus r*, where η is the viscosity of DNA in the presence of the compound, and η_0 is the viscosity of DNA alone in buffer solution.

S3.4 EB−displacement studies

The competition of the compounds with EB was investigated by fluorescence emission spectroscopy to examine whether the compounds can displace EB from its DNA−EB adduct. The CT DNA−EB complex was formed by pre−treating 40 μM EB and 46 μM CT DNA in buffer (150 mM NaCl and 15 mM trisodium citrate at pH 7.0). The possible displacement of EB by the compound sand subsequently the intercalating effect was studied by the stepwise addition of a certain amount of the solution of each compound into the solution of the CT DNA−EB adduct. The solutions were excited at 540 nm and the emission was monitored from 550−700 nm with λ_{max} = 592−594 nm and the effect of the addition of the compounds to the CT−DNA EB solution was recorded. The compounds did not display any fluorescence emission bands at room temperature in solution or in the presence of CT DNA or EB under the same experimental conditions ($\lambda_{excitation}$ = 540 nm); therefore, the observed quenching of the EB−DNA solution may be attributed to the displacement of EB from its EB−DNA adduct.

The Stern−Volmer constants (K_{SV}, in M^{-1}) were calculated according to the linear Stern−Volmer equation (equation S2) [6] and the respective plots Io/I *versus* [compound]:

$$\frac{Io}{I} = 1 + K_q\tau_0[Q] = 1 + K_{SV}[Q] \qquad \text{(equation S2)}$$

where Io and I are the emission intensities of the EB−DNA solution in the absence and the presence of the compound, respectively, τ_o = the average lifetime of the emitting system without the quencher and k_q = the quenching constant. Taking τ_o = 23 ns as the fluorescence lifetime of the EB−DNA adduct [7], the quenching constant of the compound (K_q, in $M^{-1}s^{-1}$) was calculated according to equation [6]:

$$K_{SV} = K_q \times \tau_o \qquad \text{(equation S3)}$$

S4 Plasmid DNA cleavage experiments

The reaction mixtures (20 μL) containing supercoiled circular pBR322 plasmid DNA stock solution (Form I, 50 μM/base pair, ~500 ng), compounds, and Tris buffer (25 μM, pH 6.8) in Eppendorf PCR tubes were incubated for 30 min at 37 °C and centrifuged under aerobic conditions at room temperature. Afterwards, in the experiments that irradiation was used, the reaction mixtures contained in the Eppendorf PCR tubes, where irradiated at room temperature with UVB light (312 nm) for 30 min, at a distance of 15 cm, and in the case of UVA light (365 nm) and visible light (400 nm) for 2 h, at a distance of 10 cm.

After addition of the gel−loading buffer [6x Orange DNA Loading Dye 10 mM Tris−HCl (pH 7.6), 0.15% orange G, 0.03% xylene cyanol FF, 60% glycerol, and 60 mM EDTA, by Fermentas], the reaction mixtures were loaded on a 1% agarose gel with EB staining. The electrophoresis tank was attached to a power supply at a constant current (75 V for 30 min). The gel was visualized by the Mupid−ONE LED Illuminator and photographed by a Nikon Digital Camera D3400. Quantification of DNA−cleaving activities was performed by integration of the optical density as a function of the band area using the program "Image J" available at the site http://rsb.info.nih.gov/ij/download.html.

The ss% and ds% damages were calculated according to the equations S4 and S5:

$$ss\% = \frac{Form\ II}{(Form\ I + Form\ II + Form\ III)} \times 100 \qquad \text{(equation S4)}$$

$$ds\% = \frac{Form\ III}{(Form\ I + Form\ II + Form\ III)} \times 100 \qquad \text{(equation S5)}$$

where, as Form II we consider Form II of each series minus Form II of the irradiated control DNA and as Form I, we consider Form I of each series. The amount of supercoiled DNA was multiplied by factor of 1.43 to account for reduced EB intercalation into supercoiled DNA [8].

S5 Albumin−binding studies

S5.1 Interaction with albumins

In order to study the interaction of the compounds with the albumins, the compound was initially dissolved in DMSO (1 mM). Mixing of such solutions with the aqueous buffer solutions of the albumins used in the studies never exceeded 5% DMSO (v/v) in the final solution, which was needed due to low aqueous solubility of most compounds.

With the purpose to investigate if the compound can bind to carrier protein like serum albumins, albumin binding studies were carried out by tryptophan fluorescence quenching experiments using BSA or HSA (3 μM) in buffer (containing 15 mM trisodium citrate and 150 mM NaCl at pH 7.0). The quenching of the emission intensity of tryptophan residues of BSA at 344 nm or HSA at 342 nm was monitored using the compound as quenchers with increasing concentration [6]. The fluorescence emission spectra of the compound were also recorded with $\lambda_{excitation}$ = 295 nm; in case that an additional emission band appeared the albumin−fluorescence emission spectra were corrected by subtracting the spectra of the compound. The influence of the inner−filter effect on the measurements was evaluated by the equation [9] :

$$I_{corr} = I_{meas} \times 10^{\frac{\varepsilon(\lambda_{exc})cd}{2}} \times 10^{\frac{\varepsilon(\lambda_{em})cd}{2}} \qquad \text{(equation S6)}$$

where I_{corr} = corrected intensity, I_{meas} = the measured intensity, c = the concentration of the quencher, d = the cuvette (1 cm), $\varepsilon_{(\lambda exc)}$ and $\varepsilon_{(\lambda em)}$ = the ε of the quencher at the excitation and the emission wavelength, respectively, as calculated from the UV−vis spectra of the compound [9].

The Stern−Volmer and Scatchard graphs are used to study the interaction of the compound with the albumins. According to the Stern−Volmer quenching equation (equation S2), where Io = initial tryptophan fluorescence intensity of albumin, I = tryptophan fluorescence intensity of BSA after the addition of the quencher, K_q = quenching constant, K_{SV} = Stern−Volmer constant, τ_o = average lifetime of albumin without the quencher, and, taking as fluorescence lifetime (τ_o) of tryptophan in albumin at around 10^{-8} s [6], K_{SV} (in M^{-1}) can be obtained by the slope of the diagram Io/I *versus* [compound] (Stern−Volmer plots),

and subsequently the quenching constant (K_q, in $M^{-1}s^{-1}$) may be calculated from equation S3.

From the Scatchard equation:

$$\frac{\Delta I/_{\mathrm{Io}}}{[Q]} = \mathrm{nK} - K\frac{\Delta \mathrm{I}}{\mathrm{Io}} \qquad \text{(equation S7)}$$

where n is the number of binding sites per albumin and K is the albumin–binding constant (K, in M^{-1}) is calculated from the slope in plots (ΔI/Io)/[compound] *versus* (ΔI/Io) and n is given by the ratio of y intercept to the slope [10].

S5.2 Competitive albumin–fluorescence studies with warfarin and ibuprofen

The competitive studies with warfarin or ibuprofen (as site–markers) [11] were performed by tryptophan fluorescence quenching experiments using a fixed concentration of the albumin and site markers (3 μM) in buffer (containing 15 mM trisodium citrate and 150 mM NaCl at pH 7.0). The fluorescence emission spectra were recorded in the presence of increasing amounts of the compounds as quenchers with an excitation wavelength of 295 nm. The Scatchard equation (equation S7) [10] and plots were applied on the corrected albumin–fluorescence emission spectra to determine the albumin–binding constant of the compounds in the presence of warfarin or ibuprofen.

S6 References

1. Kontogiorgis, C.; Hadjipavlou-Litina, D. Biological Evaluation of Several Coumarin Derivatives Designed as Possible Anti-Inflammatory/Antioxidant Agents. *J Enzyme Inhib Med Chem* **2003**, *18*, 63–69, doi:10.1080/1475636031000069291.
2. Ruch, R.J.; Cheng, S.; Klaunig, J.E. Prevention of Cytotoxicity and Inhibition of Intercellular Communication by Antioxidant Catechins Isolated from Chinese Green Tea. *Carcinogenesis* **1989**, *10*, 1003–1008, doi:10.1093/CARCIN/10.6.1003.
3. Andrews, J.M. Determination of Minimum Inhibitory Concentrations. *Journal of Antimicrobial Chemotherapy* **2001**, *48*, 5–16, doi:10.1093/JAC/48.SUPPL_1.5.
4. Irgi, E.P.; Geromichalos, G.D.; Balala, S.; Kljun, J.; Kalogiannis, S.; Papadopoulos, A.; Turel, I.; Psomas, G. Cobalt(II) Complexes with the Quinolone Antimicrobial Drug Oxolinic Acid: Structure and Biological Perspectives. *RSC Adv* **2015**, *5*, 36353–36367, doi:10.1039/c5ra05308k.
5. Wolfe, A.; Shimer, G.H.; Meehan, T. Polycyclic Aromatic Hydrocarbons Physically Intercalate into Duplex Regions of Denatured DNA. *Biochemistry* **1987**, *26*, 6392–6396, doi:10.1021/bi00394a013.
6. Lakowicz, J.R. *Principles of Fluorescence Spectroscopy*; Springer, 2006; ISBN 0387312781.
7. Heller, D.P.; Greenstock, C.L. Fluorescence Lifetime Analysis of DNA Intercalated Ethidium Bromide and Quenching by Free Dye. *Biophys Chem* **1994**, *50*, 305–312, doi:10.1016/0301-4622(93)E0101-A.
8. Papastergiou, A.; Perontsis, S.; Gritzapis, P.; Koumbis, A.E.; Koffa, M.; Psomas, G.; Fylaktakidou, K.C. Evaluation of O-Alkyl and Aryl Sulfonyl Aromatic and Heteroaromatic Amidoximes as Novel Potent DNA Photo-Cleavers. *Photochemical and Photobiological Sciences* **2016**, *15*, 351–360, doi:10.1039/c5pp00439j.
9. Stella, L.; Capodilupo, A.L.; Bietti, M. A Reassessment of the Association between Azulene and [60]Fullerene. Possible Pitfalls in the Determination of Binding Constants through Fluorescence Spectroscopy. *Chemical Communications* **2008**, 4744–4746, doi:10.1039/b808357f.
10. Wang, Y.-Q.; Zhang, H.-M.; Zhang, G.-C.; Tao, W.-H.; Tang, S.-H. Interaction of the Flavonoid Hesperidin with Bovine Serum Albumin: A Fluorescence Quenching Study. *J Lumin* **2007**, *126*, 211–218, doi:10.1016/J.JLUMIN.2006.06.013.
11. Lazou, M.; Tarushi, A.; Gritzapis, P.; Psomas, G. Transition Metal Complexes with a Novel Guanine-Based (E)-2-(2-(Pyridin-2-Ylmethylene)Hydrazinyl)Quinazolin-4(3H)-One: Synthesis, Characterization, Interaction with DNA and Albumins and Antioxidant Activity. *J Inorg Biochem* **2020**, *206*, 111019, doi:10.1016/j.jinorgbio.2020.111019.
12. de Meulenaer, J.; Tompa, H. The Absorption Correction in Crystal Structure Analysis. *Acta Crystallogr* **1965**, *19*, 1014–1018, doi:10.1107/S0365110X65004802.

TABLES

Table S1. Experimental crystallographic details for complex **4**.

**Crystal data**	
Chemical formula	$C_{56}H_{48}Co_2N_8O_{10}$
M_r	1110.91
Crystal system, space group	Triclinic, $P\bar{1}$
Temperature (K)	295
a, *b*, *c* (Å)	8.3973 (17), 12.647 (3), 13.317 (3)
α, β, γ (°)	68.615 (8), 72.649 (8), 74.349 (8)
V (Å^3)	1236.1 (4)
Z	1
Radiation type	Mo *K*α
μ (mm^{-1})	0.74
Crystal size (mm)	0.24 × 0.11 × 0.06
**Data collection**	
Diffractometer	Bruker Kappa Apex2
Absorption correction	Numerical<br>Analytical Absorption [12]
T_{min}, T_{max}	0.92, 0.96
No. of measured reflections	18583
No. of independent reflections	4840
No. of observed [$I > 2.0\sigma(I)$] reflections	4074
R_{int}	0.029
$(\sin \theta/\lambda)_{max}$ (Å^{-1})	0.620
**Refinement**	
$R[F^2 > 2\sigma(F^2)]$, $wR(F^2)$, S	0.035, 0.065, 1.00
No. of reflections	4074
No. of parameters	343
H–atom treatment	H–atom parameters constrained
$\Delta\rho_{max}$, $\Delta\rho_{min}$ (e Å^{-3})	0.39, −0.32

FIGURES

Figure S1. ^{1}H NMR spectrum of 4′–NO_2–fenH in DMSO-d_6.

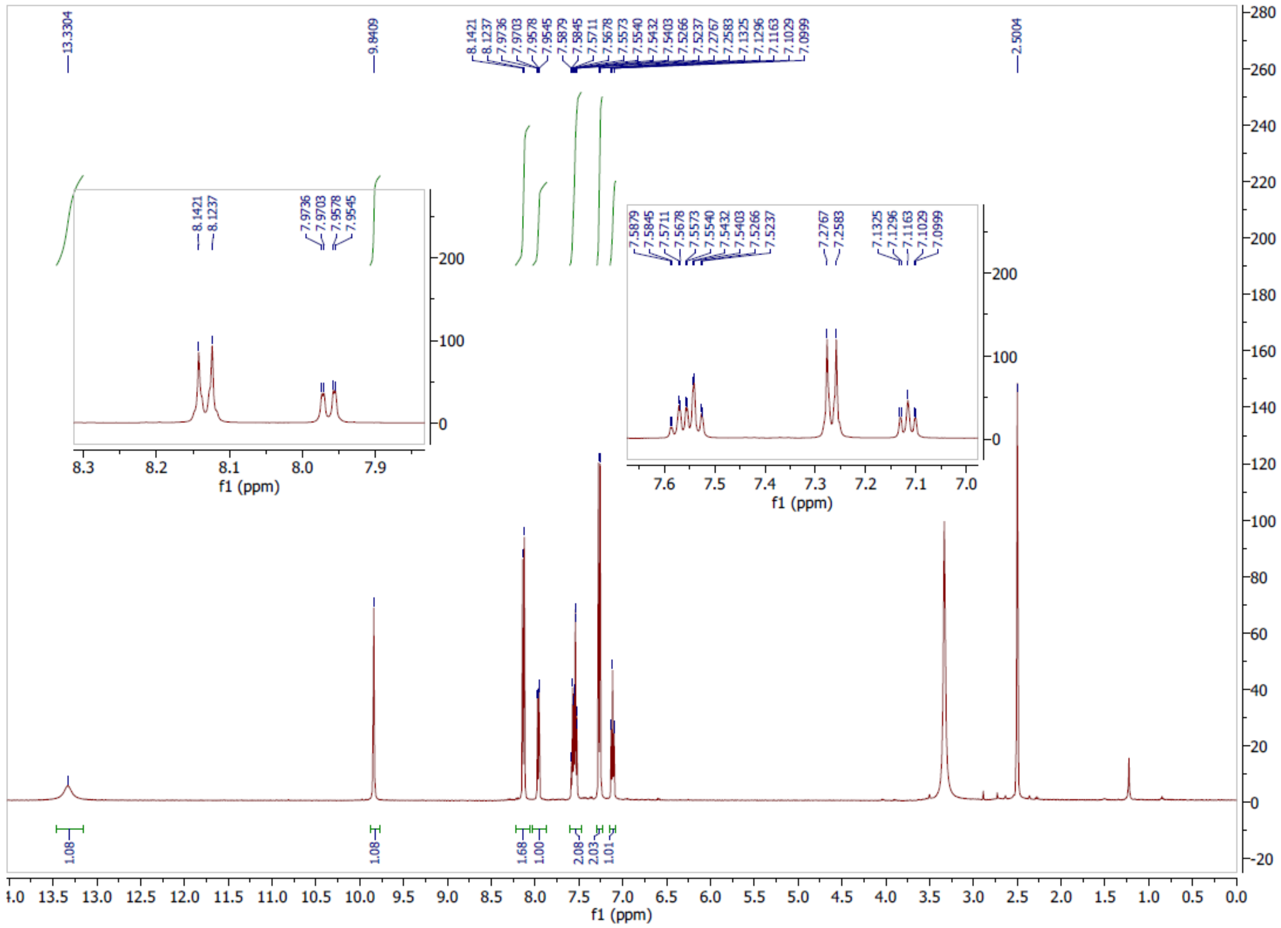

Figure S2. IR spectra (ATR) of the compounds.

<table>
<tr><td>4′–NO2–fenH</td><td>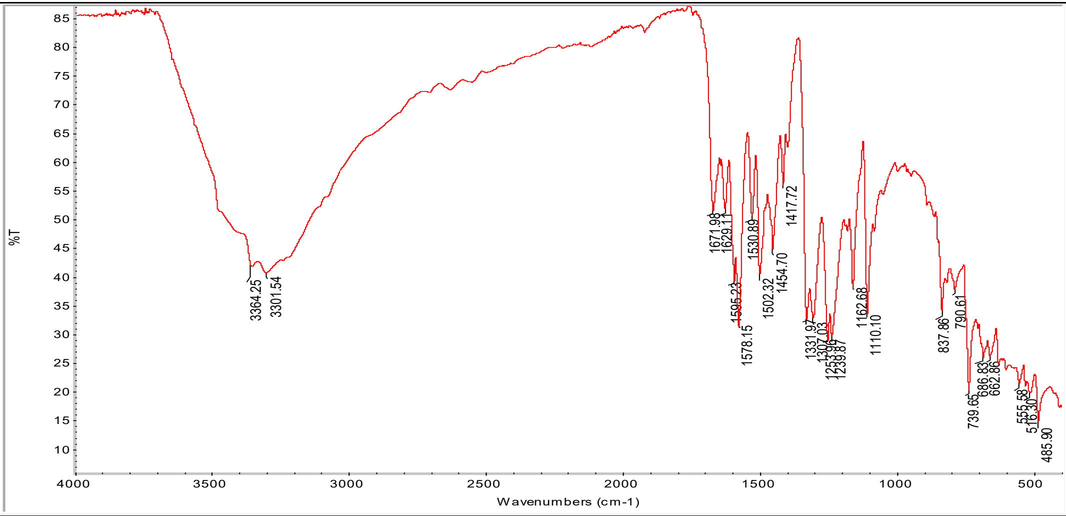
</td></tr>
<tr><td>Complex **1**</td><td>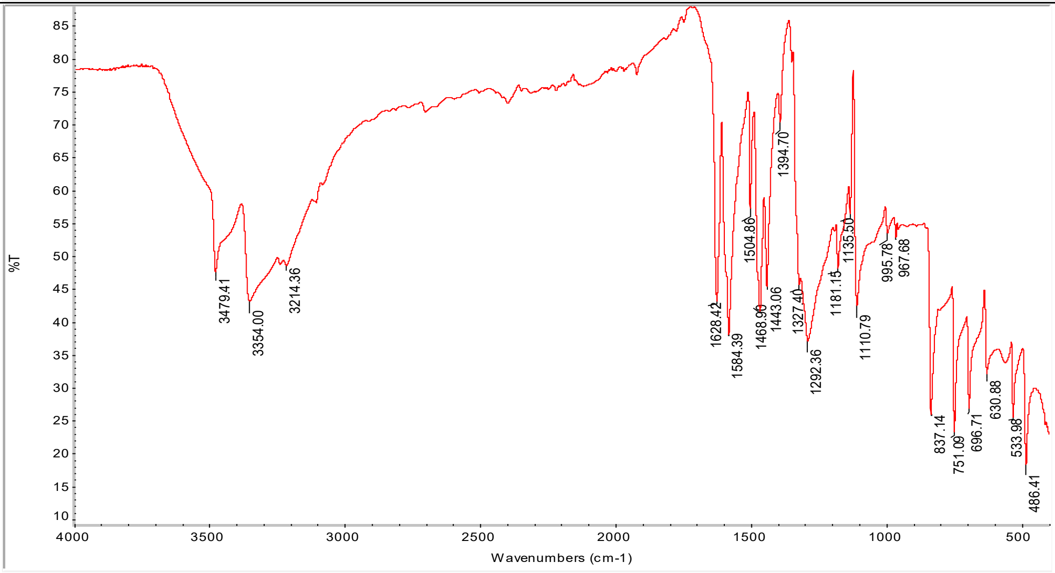
</td></tr>
<tr><td>Complex **2**</td><td>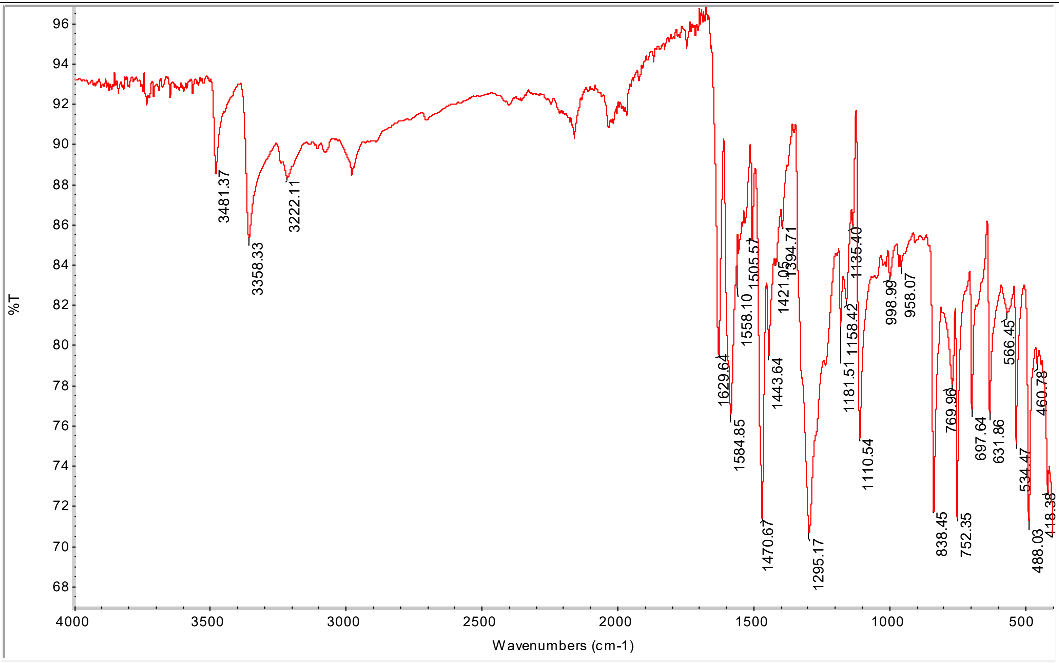
</td></tr>
</table>

<table>
<tr><td>Complex **3**</td><td>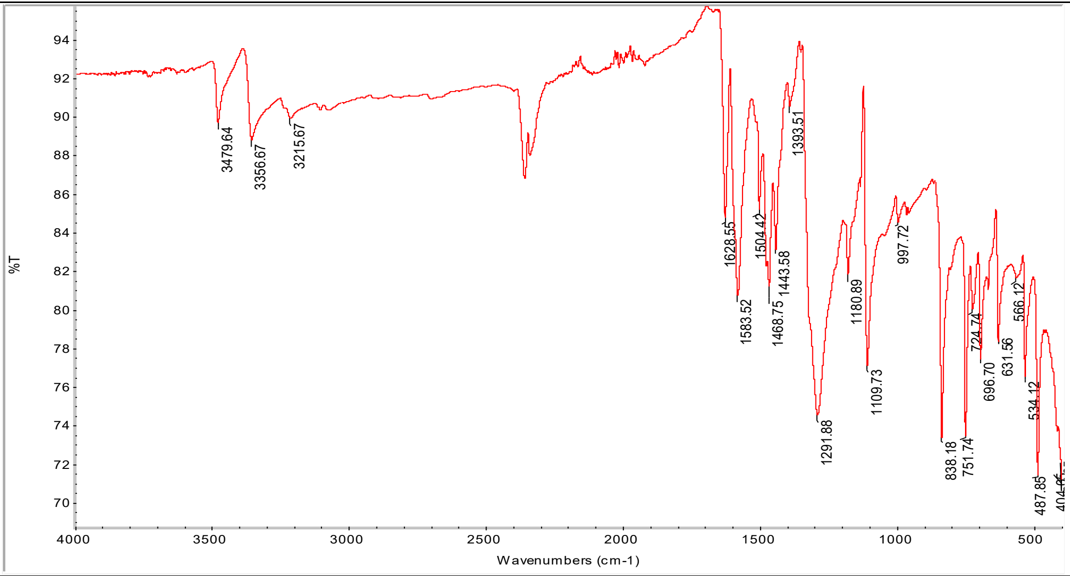
</td></tr>
<tr><td>Complex **4**</td><td>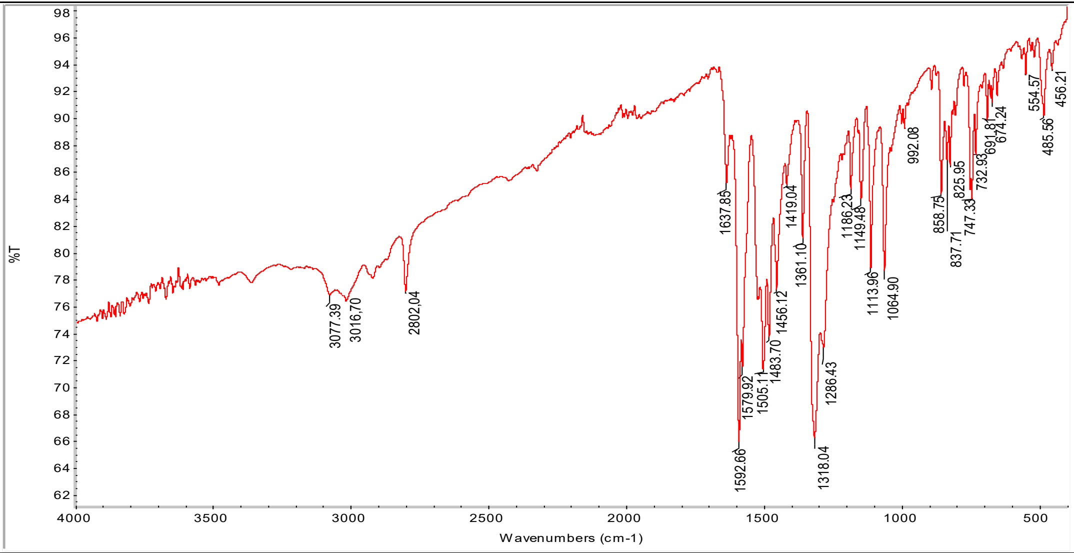
</td></tr>
</table>

Figure S3: Proposed structures for complexes **1–3**.

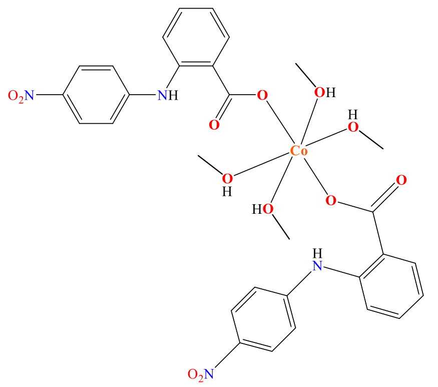	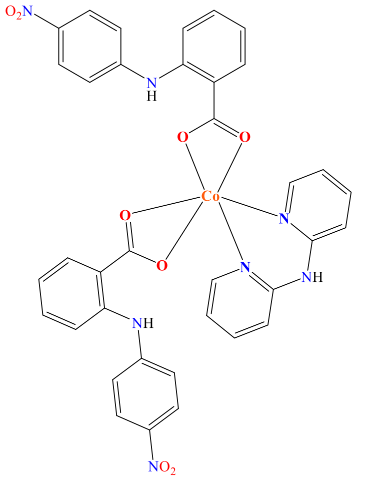	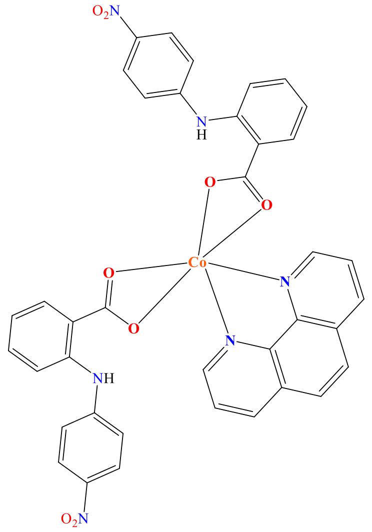
Complex **1**	Complex **2**	Complex **3**

Figure S4. UV−vis spectra of a DMSO solution of the compounds in the presence of increasing amounts of CT DNA.

The concentrations of the solution of the compounds are given in parentheses. The arrows show the changes upon increasing amounts of CT DNA.

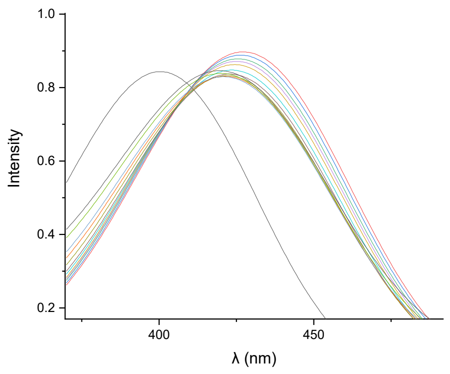

4′−NO_2−fenH (100 μM)

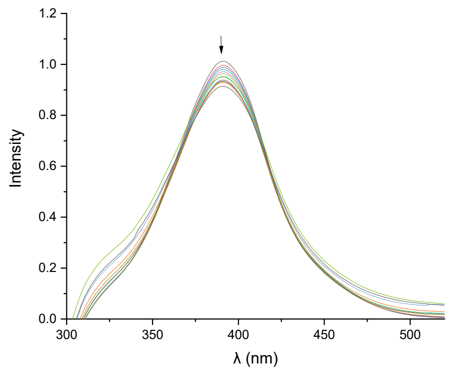

Complex **1** (20 μM)

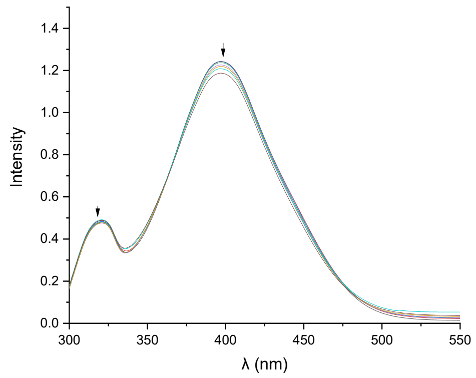

Complex **2** (30 μM)

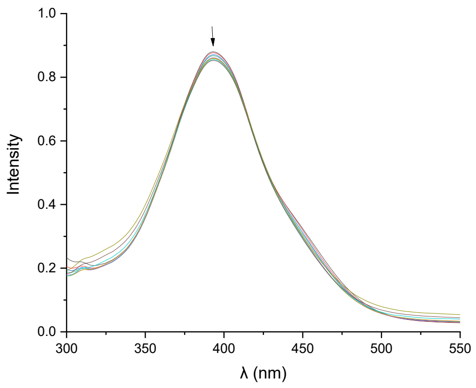

Complex **3** (20 μM)

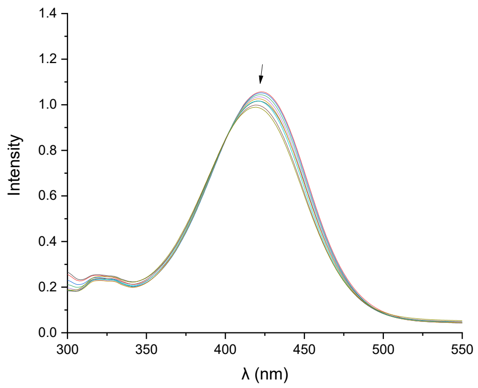

Complex **4** (20 μM)

Figure S5. Plots of $\frac{[\text{DNA}]}{(\varepsilon_A-\varepsilon_f)}$ *versus* [DNA] for the compounds.

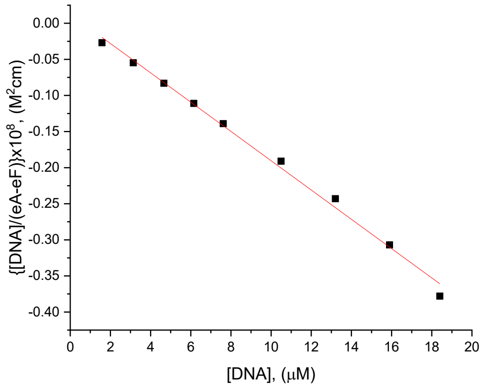

4′–NO_2–fenH

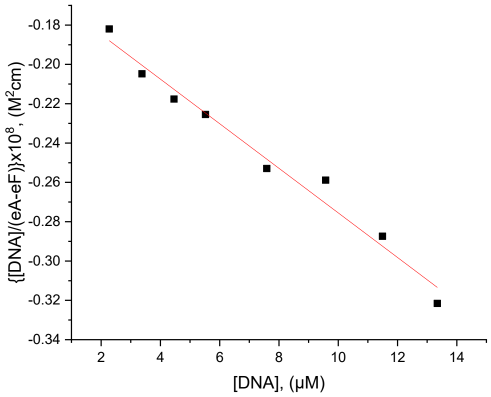

Complex **1**

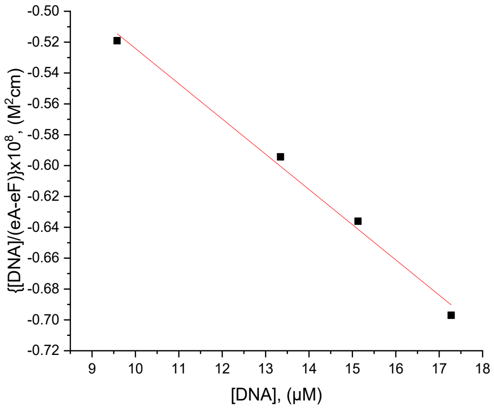

Complex **2**

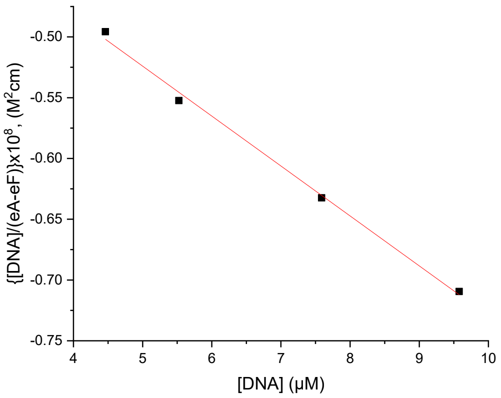

Complex **3**

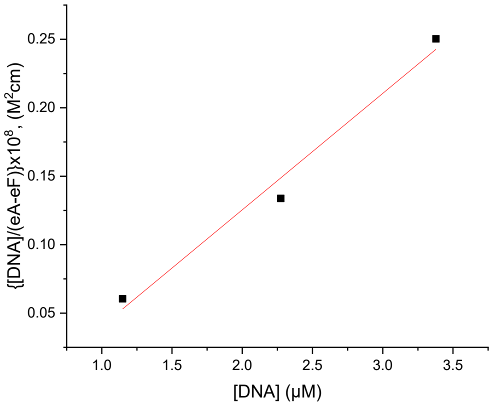

Complex **4**

Figure S6. Thermal melting profile of CT DNA in the absence or presence of the compounds.

[CT DNA] = 1.25×10^{-4} M; [Compound] = 2.5×10^{-6} M; Compound : DNA ratio = 1:50.

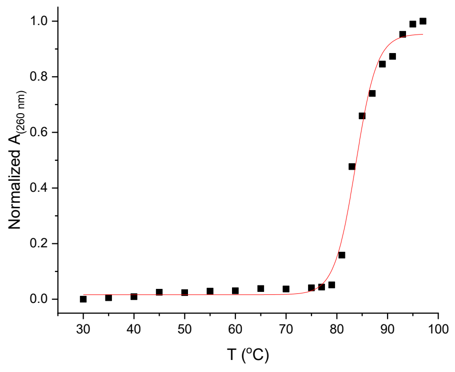

4′–NO_2–fenH

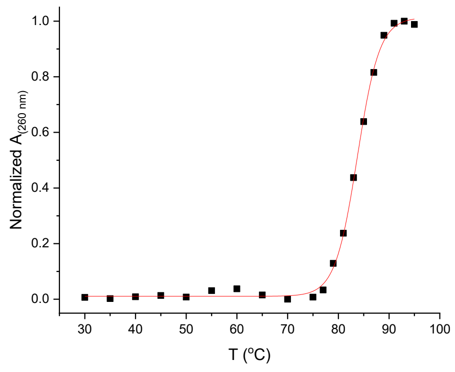

Complex **1**

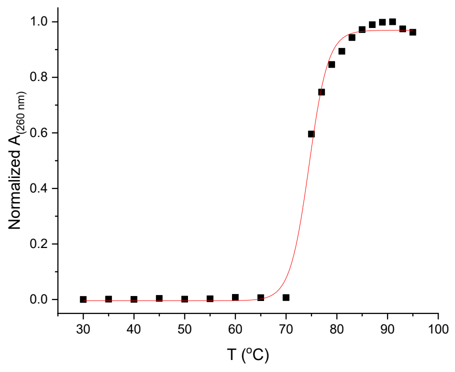

Complex **2**

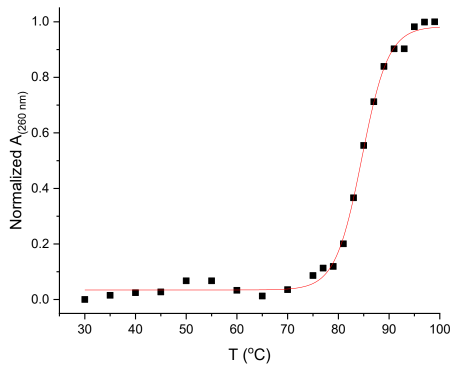

Complex **3**

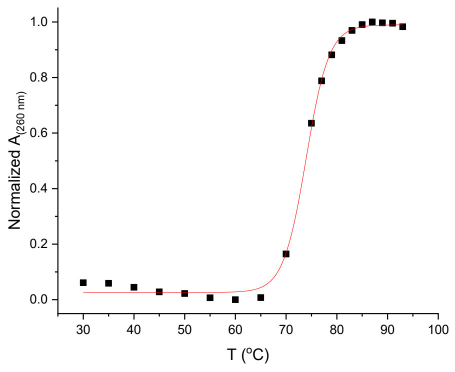

Complex **4**

Figure S7. Fluorescence emission spectra for EB–DNA in buffer solution in the absence and presence of increasing amounts of the compounds.

Conditions: $\lambda_{excitation}$ = 540 nm. [EB] = 40 μM. [DNA] = 45 μM. Buffer solution: 150 mM NaCl and 15 mM trisodium citrate at pH 7.0.

The arrow shows the changes of intensity upon increasing amounts of the compound.

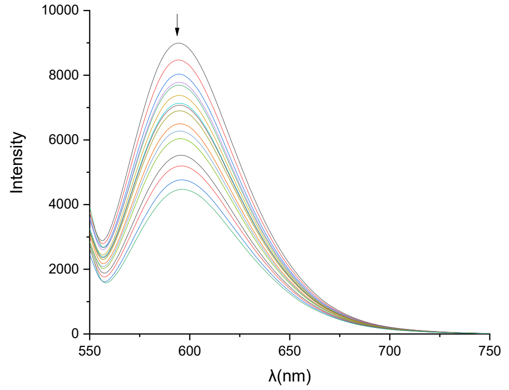

4′–NO_2–fenH

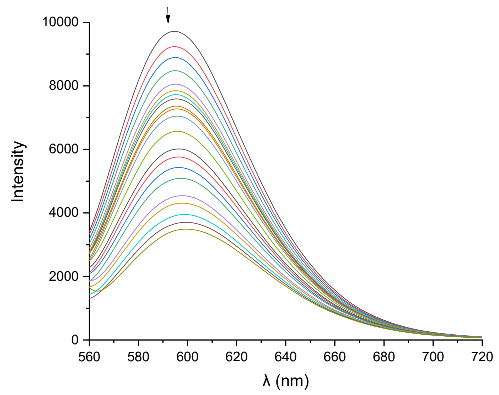

Complex **1**

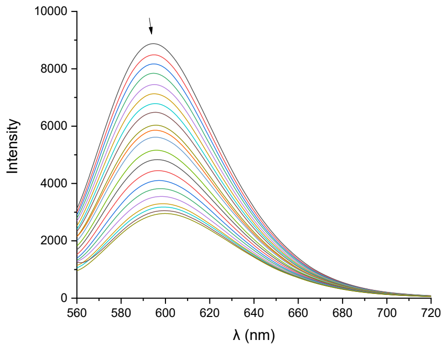

Complex **2**

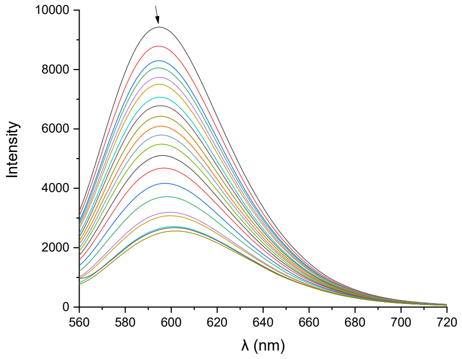

Complex **3**

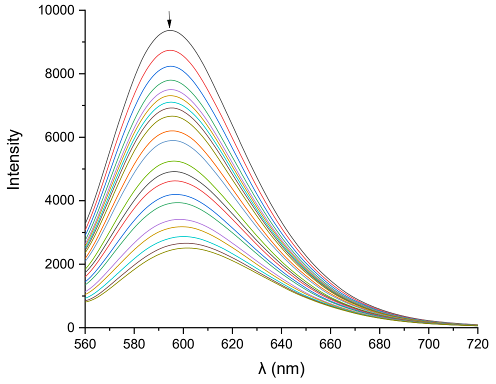

Complex **4**

Figure S8. Stern–Volmer plots of the EB–DNA quenching experiments upon addition of the compounds.

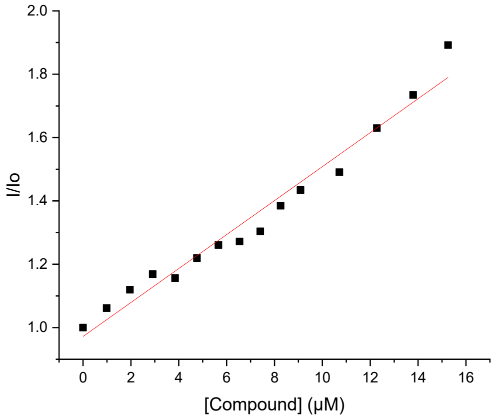

4′–NO_2–fenH

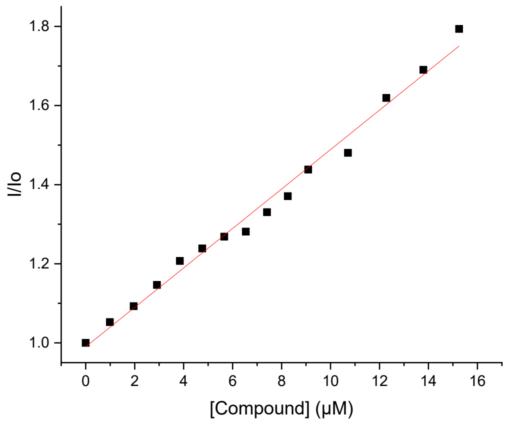

Complex **1**

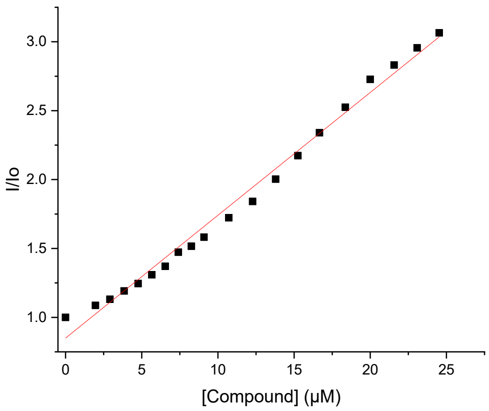

Complex **2**

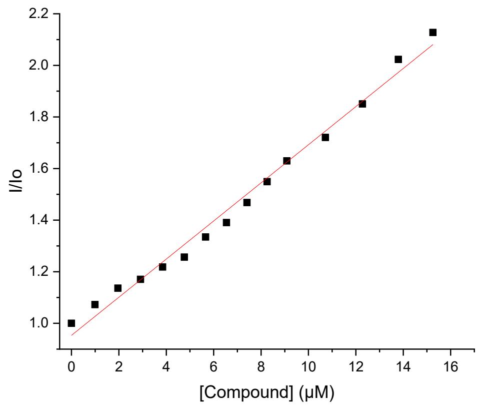

Complex **3**

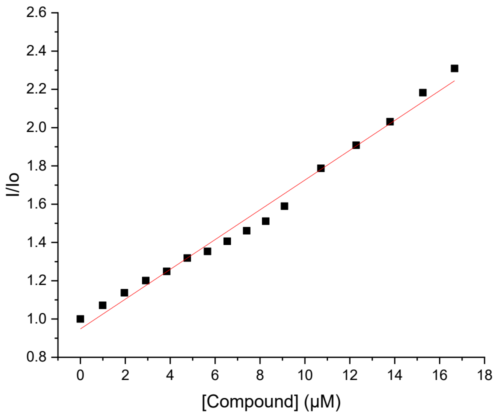

Complex **4**

Figure S9: Agarose gel electrophoretic pattern of EB–stained plasmid DNA (pBR322 plasmid DNA) after incubation with the compounds, in dark.

Conditions: [pDNA] = 50 μM/base pair. [compound] = 500 μM. Power supply: 65 V for 1 h.

Top: gel electrophoresis pictures: lane 1: complex **1** + pDNA; lane 2: complex **2** + pDNA; lane 3: complex **3** + pDNA; lane 4: complex **4** + pDNA; lane 5: 4′–NO_2–fenH + pDNA; lane 6: pDNA.

Bottom: calculation of the % conversion to ss and ds damage. DNA forms: form I = supercoiled pDNA, form II = relaxed pDNA, and form III = linear pDNA.

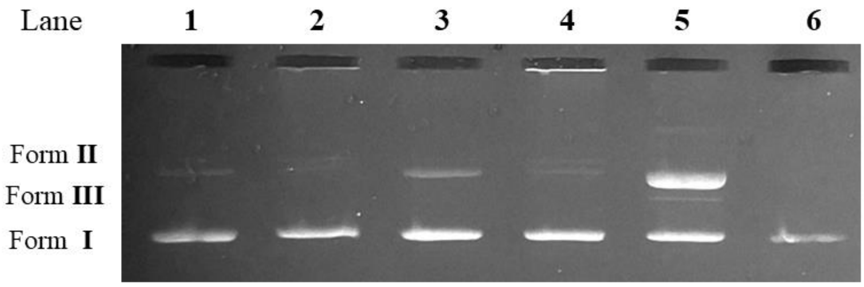

Form **II**	**17**	**16**	**29**	**19**	**46**	
Form **III**	**0**	**0**	**0**	**0**	**11**	

Figure S10: Agarose gel electrophoretic pattern of EB–stained plasmid DNA (pBR322 plasmid DNA) with the compounds, after irradiation at 312 nm (UV–B) for 30 min.

Conditions: [pDNA] = 50 μM/base pair; [compound] = 500 μM. Power supply: 65 V for 1 h. Top: gel electrophoresis pictures: lane 1: complex **1** + pDNA+ irradiation; lane 2: complex **2** + pDNA+ irradiation; lane 3: complex **3** + pDNA+ irradiation; lane 4: complex **4** + pDNA+ irradiation; lane 5: 4′–NO_2–fenH + pDNA+ irradiation; lane 6: pDNA + irradiation.

Bottom: calculation of the % conversion to ss and ds damage. DNA forms: form I = supercoiled pDNA, form II = relaxed pDNA, and form III = linear pDNA.

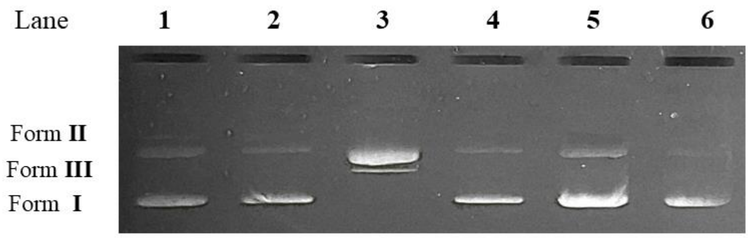

Form **II**	**40**	**27**	**73**	**33**	**24**
Form **III**	**0**	**0**	**16**	**0**	**0**

Figure S11: Agarose gel electrophoretic pattern of EB–stained plasmid DNA (pBR322 plasmid DNA) with the compounds, after irradiation at 365 nm (UV–A) for 30 min.

Conditions: [pDNA] = 50 μM/base pair; [compound] = 500 μM. Power supply: 65 V for 1 h. Top: gel electrophoresis pictures: lane 1: complex **1** + pDNA+ irradiation; lane 2: complex **2** + pDNA+ irradiation; lane 3: complex **3** + pDNA+ irradiation; lane 4: complex **4** + pDNA+ irradiation; lane 5: 4′–NO_2–fenH + pDNA+ irradiation; lane 6: pDNA + irradiation.

Bottom: calculation of the % conversion to ss and ds damage. DNA forms: form I = supercoiled pDNA, form II = relaxed pDNA, and form III = linear pDNA.

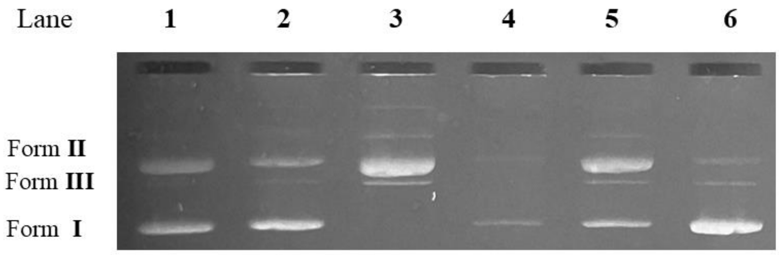

Form **II**	**37**	**30**	**62**	**35**	**36**	
Form **III**	**0**	**13**	**13**	**0**	**9**	

Figure S12: Agarose gel electrophoretic pattern of EB–stained plasmid DNA (pBR322 plasmid DNA) with the compounds, after irradiation under visible light for 2 h.

Conditions: [pDNA] = 50 μM/base pair; [compound] = 500 μM. Power supply: 65 V for 1 h. Top: gel electrophoresis pictures: lane 1: complex **1** + pDNA+ irradiation; lane 2: complex **2** + pDNA+ irradiation; lane 3: complex **3** + pDNA+ irradiation; lane 4: complex **4** + pDNA+ irradiation; lane 5: 4′–NO_2–fenH + pDNA+ irradiation; lane 6: pDNA + irradiation. Bottom: calculation of the % conversion to ss and ds damage. DNA forms: form I = supercoiled pDNA, form II = relaxed pDNA, and form III = linear pDNA.

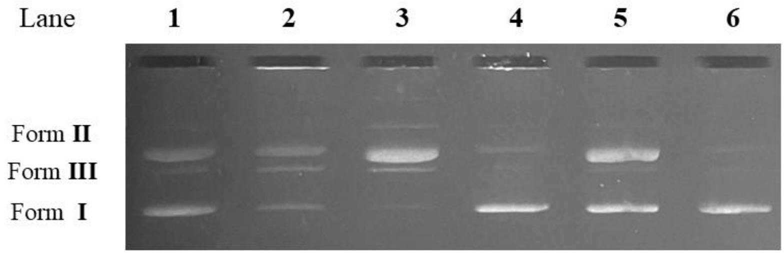

Form **II**	**33**	**37**	**60**	**22**	**40**	
Form **III**	**13**	**24**	**22**	**0**	**14**	

Figure S13. Fluorescence emission spectra of BSA in buffer solution in the presence of increasing amounts of the compounds.

Conditions: $\lambda_{excitation}$ = 295 nm. [BSA] = 3 μM. Buffer solution: 150 mM NaCl and 15 mM trisodium citrate at pH 7.0.

The arrow shows the changes of intensity upon increasing amounts of the compound.

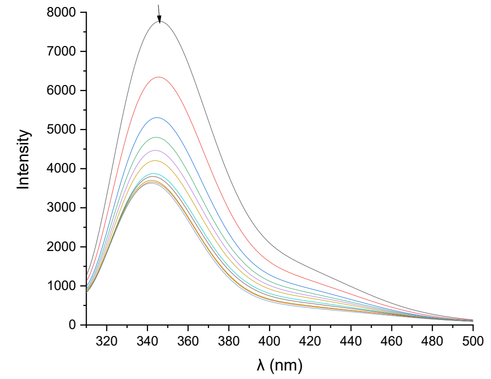

4′–NO$_2$–fenH

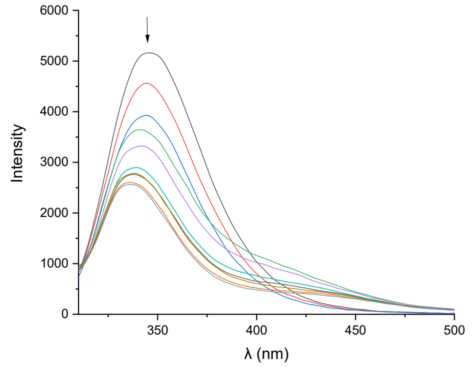

Complex **1**

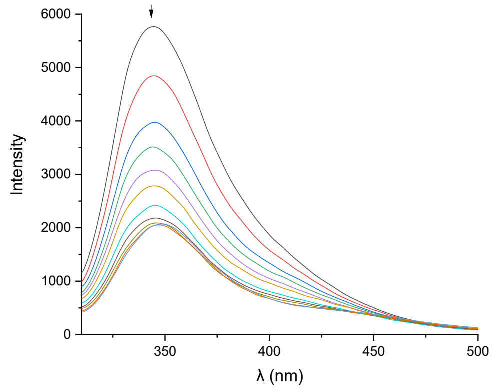

Complex **2**

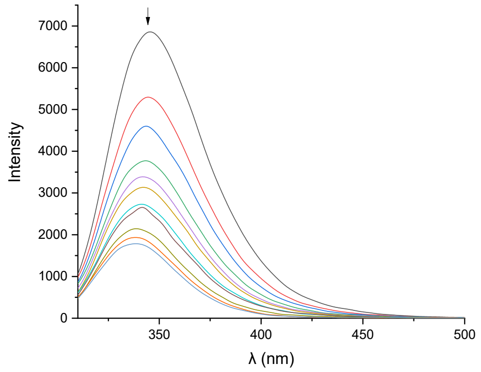

Complex **3**

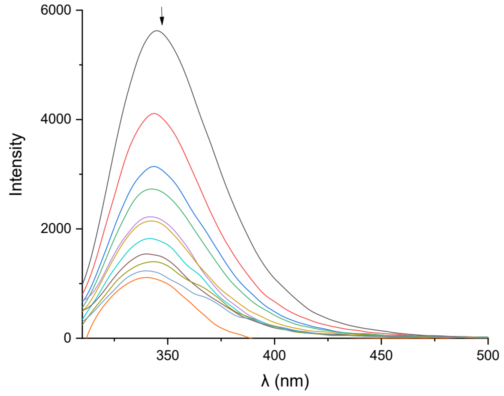

Complex **4**

Figure S14. Fluorescence emission spectra of HSA in buffer solution in the presence of increasing amounts of the compounds.

Conditions: $\lambda_{excitation}$= 295 nm. [HSA] = 3 μM. Buffer solution: 150 mM NaCl and 15 mM trisodium citrate at pH 7.0.

The arrow shows the changes of intensity upon increasing amounts of the compound.

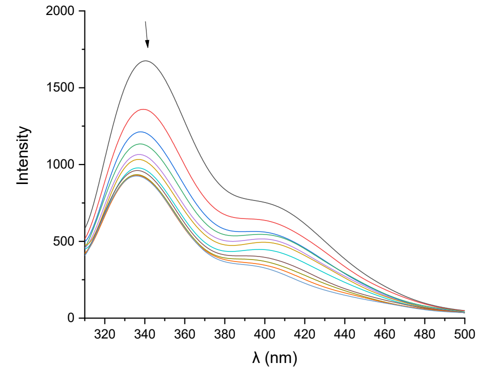	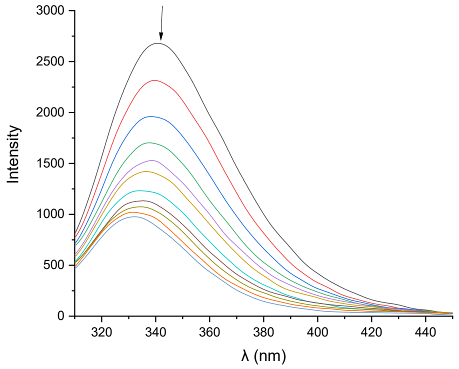
4′–NO_2–fenH	Complex **1**
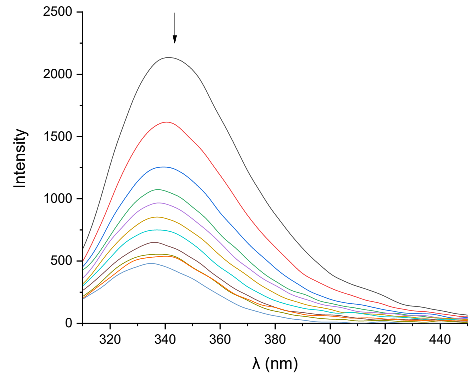	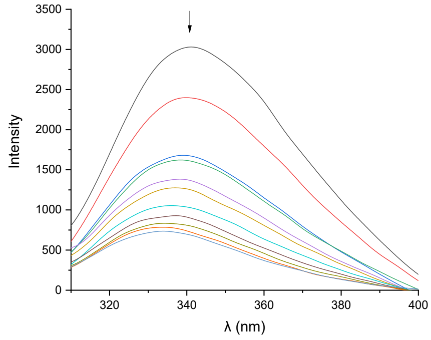
Complex **2**	Complex **3**
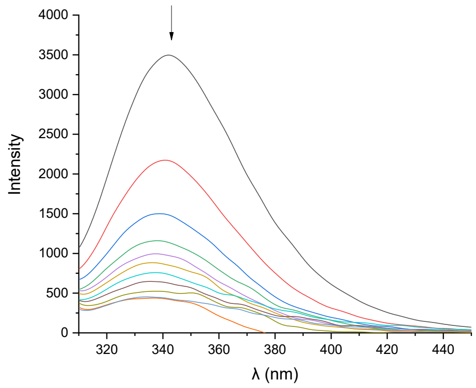	
Complex **4**	

Figure S15. Stern−Volmer plots of the BSA−quenching experiments upon addition of the compounds.

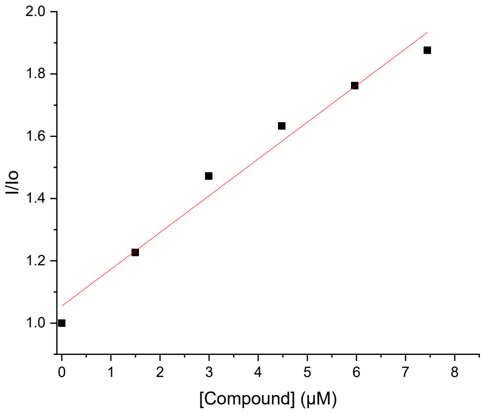

4′–NO_2–fenH

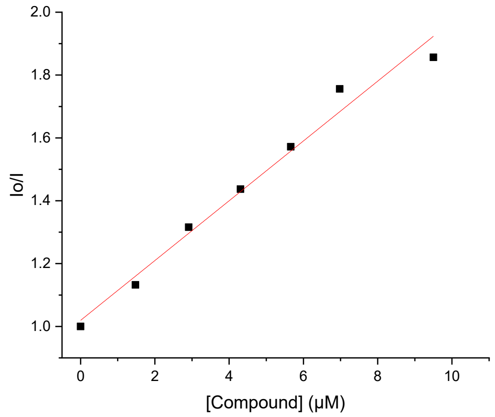

Complex **1**

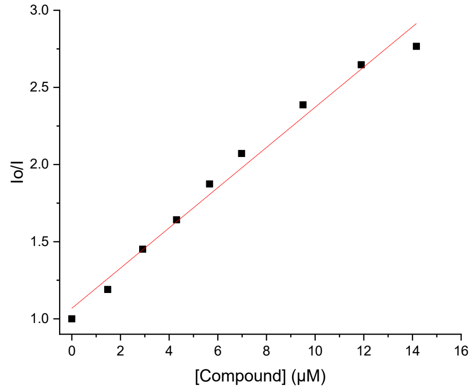

Complex **2**

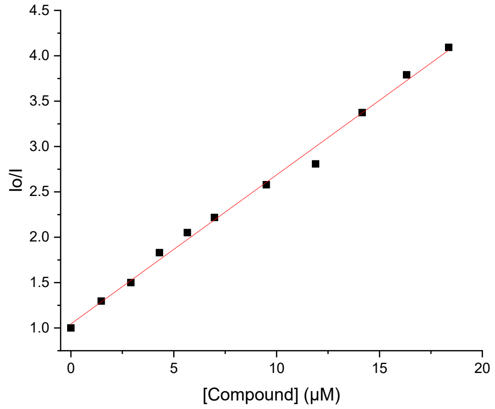

Complex **3**

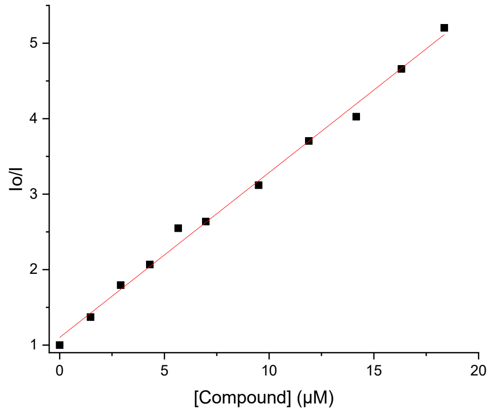

Complex **4**

Figure S16. Stern–Volmer plots of the HSA–quenching experiments upon addition of the compounds.

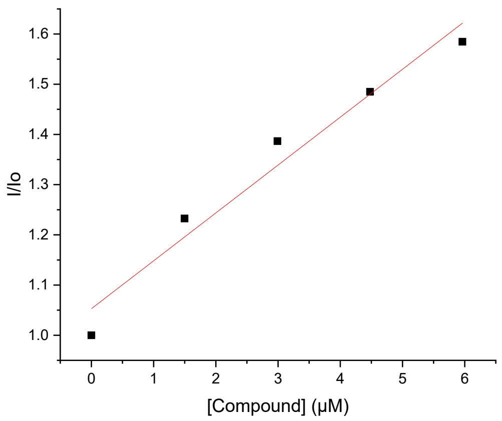

4′–NO_2–fenH

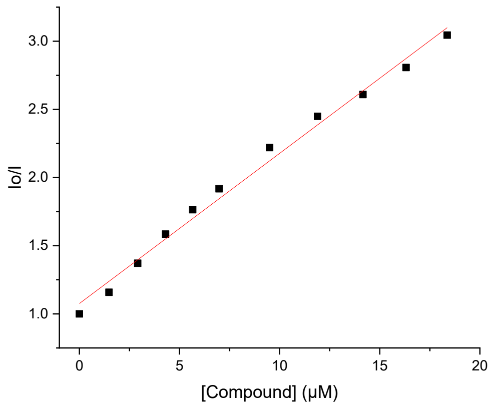

Complex **1**

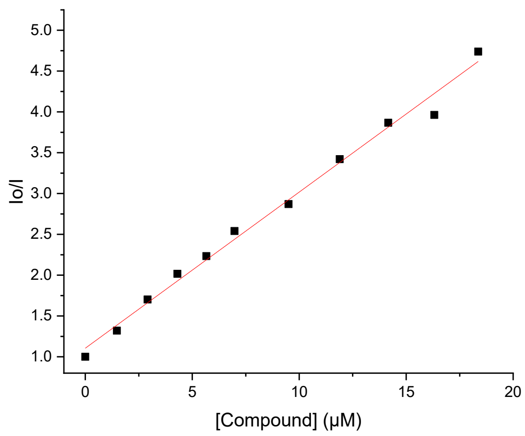

Complex **2**

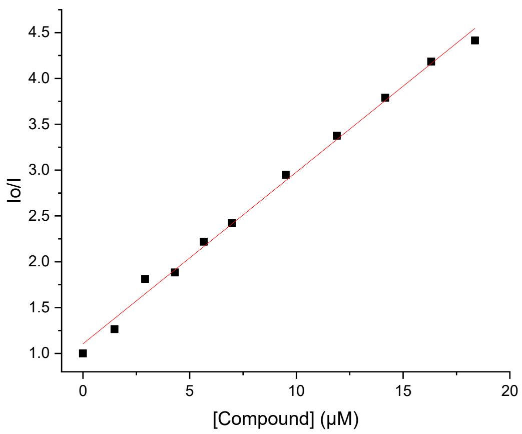

Complex **3**

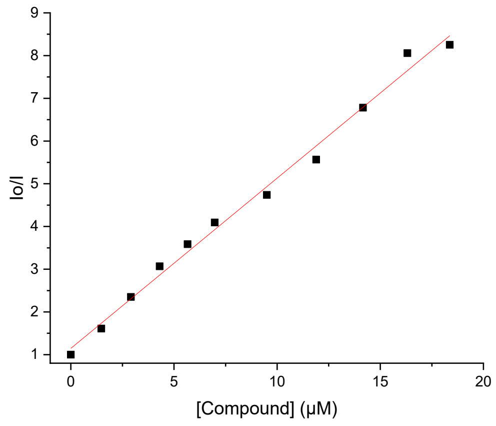

Complex **4**

Figure S17. Scatchard plots of the BSA–quenching experiments upon addition of the compounds.

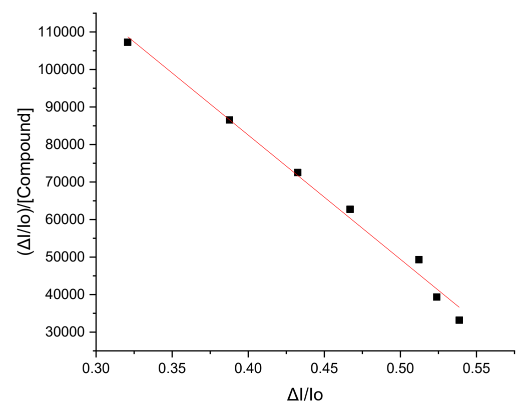

4′–NO_2–fenH

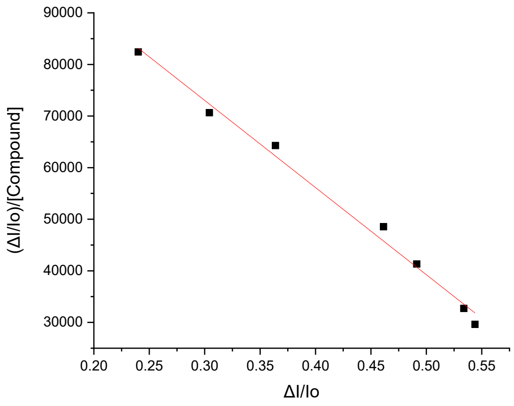

Complex **1**

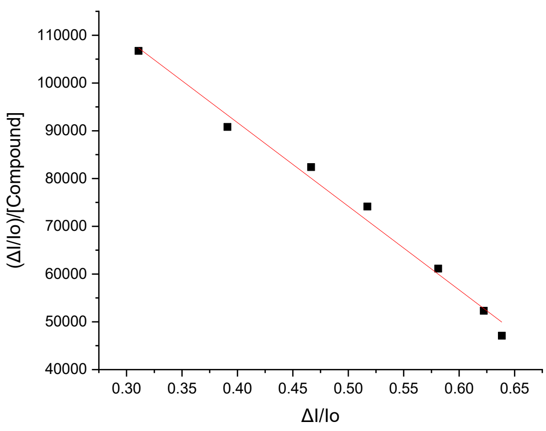

Complex **2**

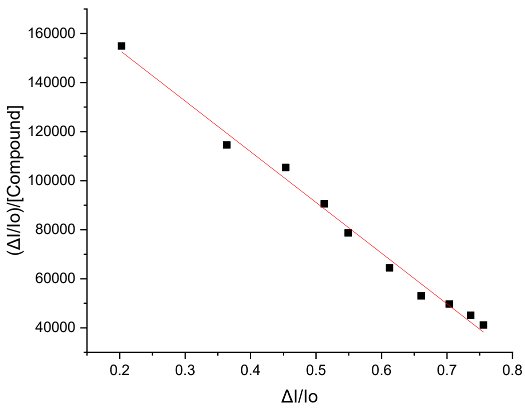

Complex **3**

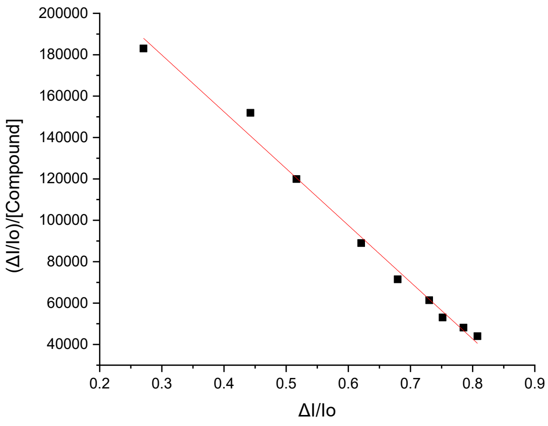

Complex **4**

Figure S18. Scatchard plots of the HSA–quenching experiments upon addition of the compounds.

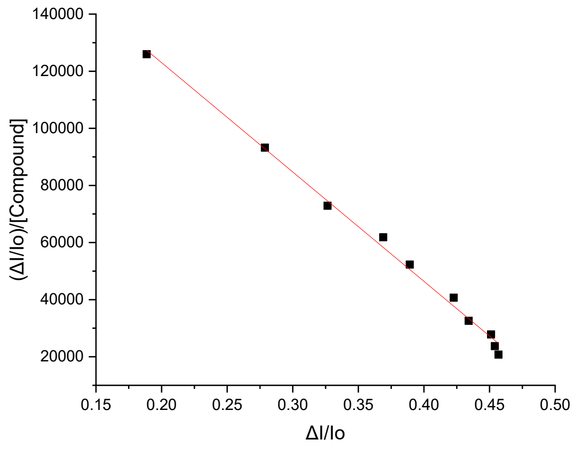

4′–NO_2–fenH

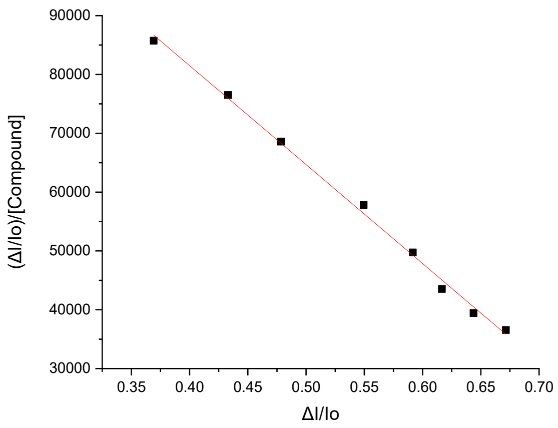

Complex **1**

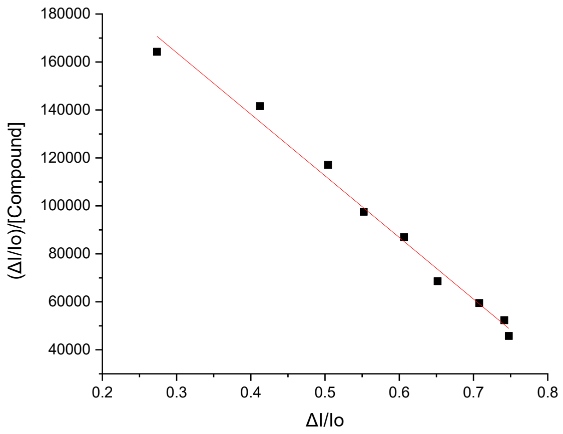

Complex **2**

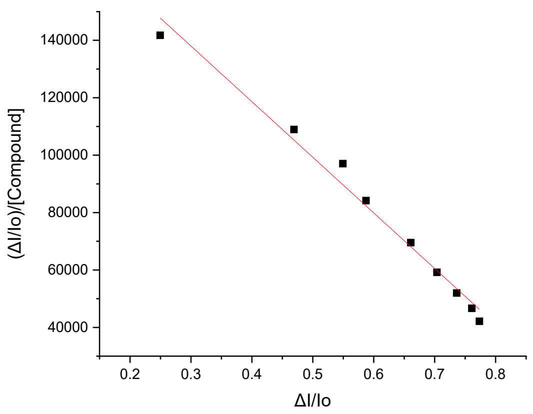

Complex **3**

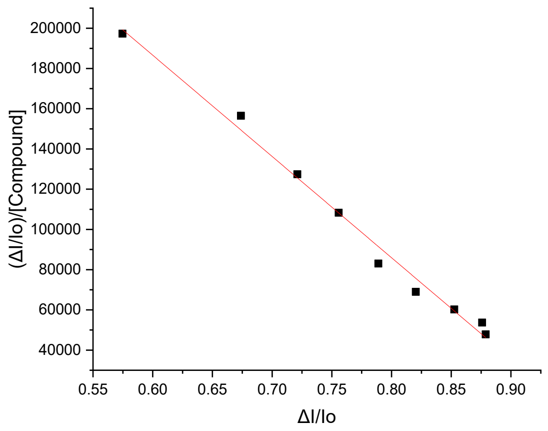

Complex **4**

Figure S19. Fluorescence emission spectra of BSA in buffer solution in the presence of ibuprofen upon addition of increasing amounts of the compounds.
Conditions: $\lambda_{excitation}$ = 295 nm. [BSA] = 3 μM. Buffer solution: 150 mM NaCl and 15 mM trisodium citrate at pH 7.0. [Ibuprofen] = 3 μM.
The arrow shows the changes of intensity upon increasing amounts of the compound.

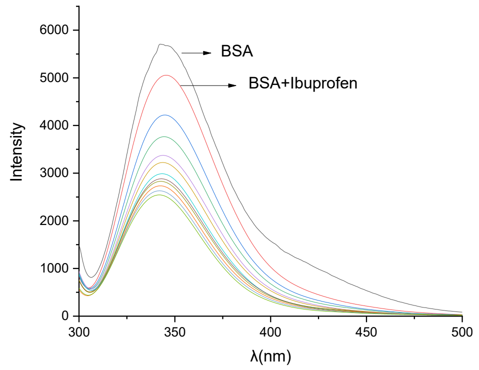

4′–NO2–fenH

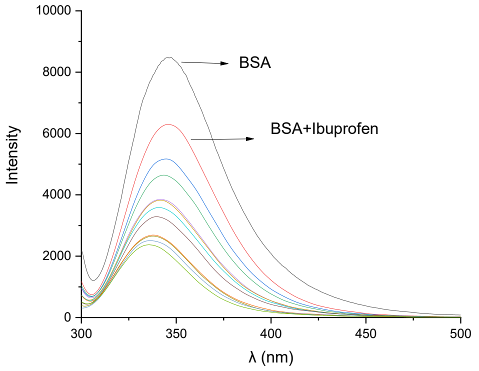

Complex **1**

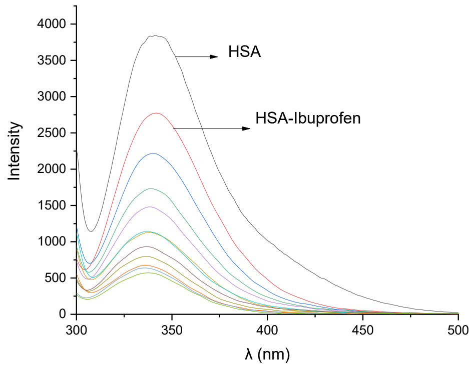

Complex **2**

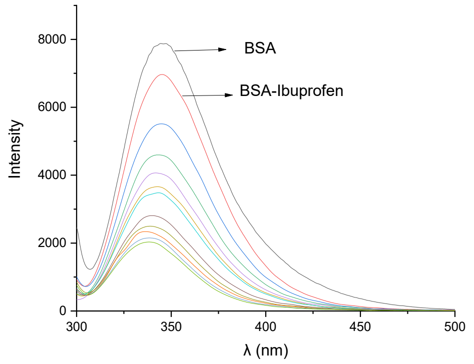

Complex **3**

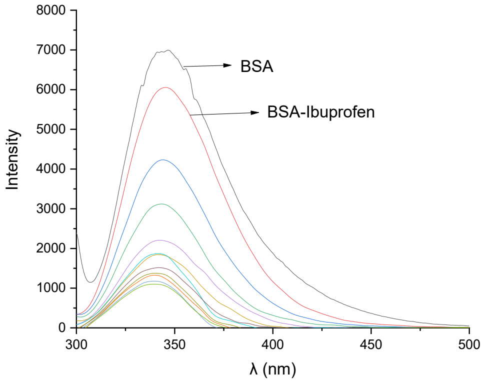

Complex **4**

Figure S20. Fluorescence emission spectra of BSA in buffer solution in the presence of warfarin upon addition of increasing amounts of the compounds.

Conditions: $\lambda_{excitation}$= 295 nm. [BSA] = 3 μM. Buffer solution: 150 mM NaCl and 15 mM trisodium citrate at pH 7.0. [Warfarin] = 3 μM.

The arrow shows the changes of intensity upon increasing amounts of the compound.

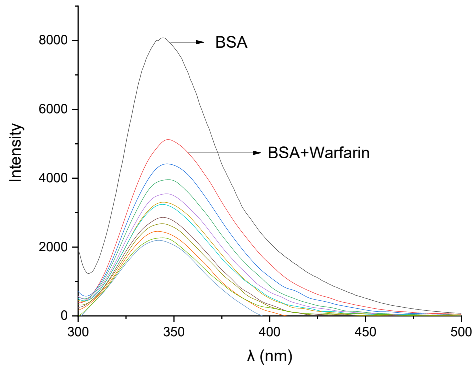

4′–NO_2–fenH

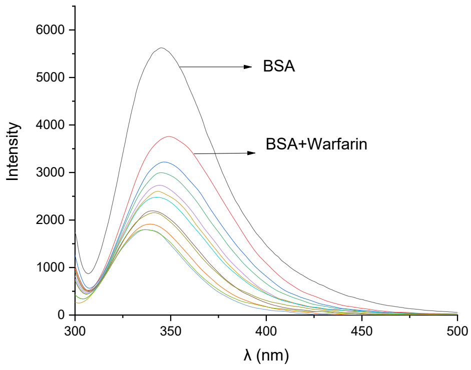

Complex **1**

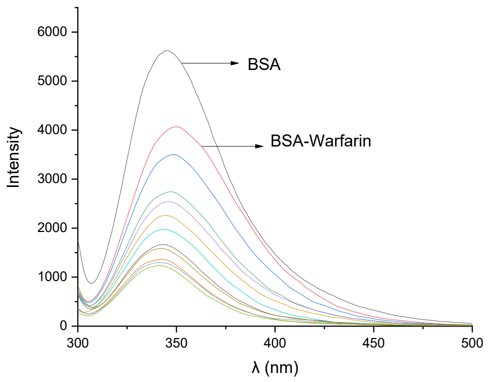

Complex **2**

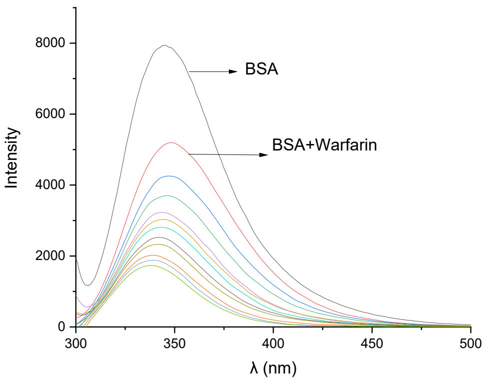

Complex **3**

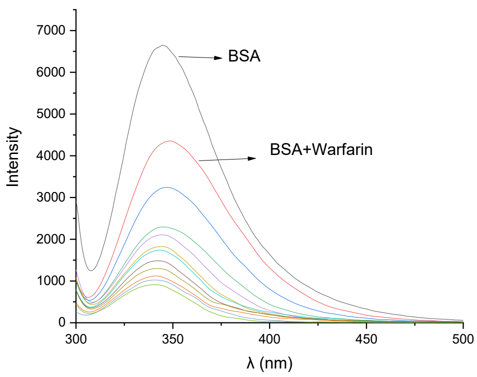

Complex **4**

Figure S21. Fluorescence emission spectra of HSA in buffer solution in the presence of ibuprofen upon addition of increasing amounts of the compounds.

Conditions: $\lambda_{excitation}$ = 295 nm. [HSA] = 3 μM. Buffer solution: 150 mM NaCl and 15 mM trisodium citrate at pH 7.0. [Ibuprofen] = 3 μM.

The arrow shows the changes of intensity upon increasing amounts of the compound.

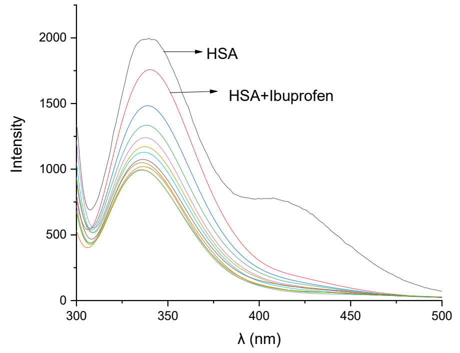

4′–NO_2–fenH

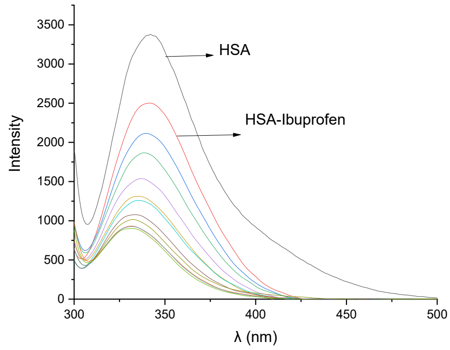

Complex **1**

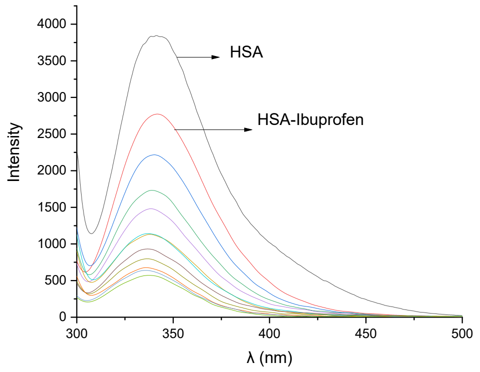

Complex **2**

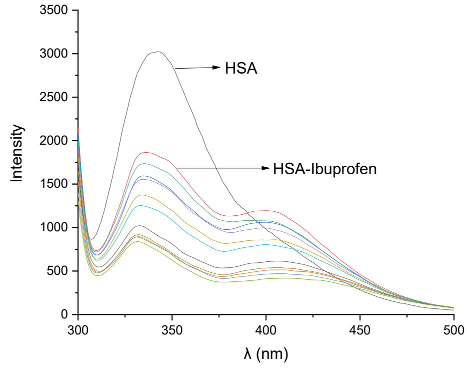

Complex **3**

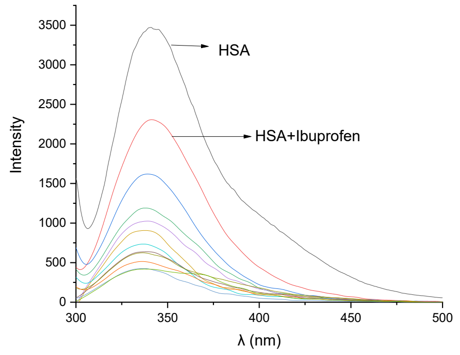

Complex **4**

Figure S22. Fluorescence emission spectra of HSA in buffer solution in the presence of warfarin upon addition of increasing amounts of the compounds. Conditions: $\lambda_{excitation}$ = 295 nm. [HSA] = 3 μM. Buffer solution: 150 mM NaCl and 15 mM trisodium citrate at pH 7.0. [Warfarin] = 3 μM.
The arrow shows the changes of intensity upon increasing amounts of the compound.

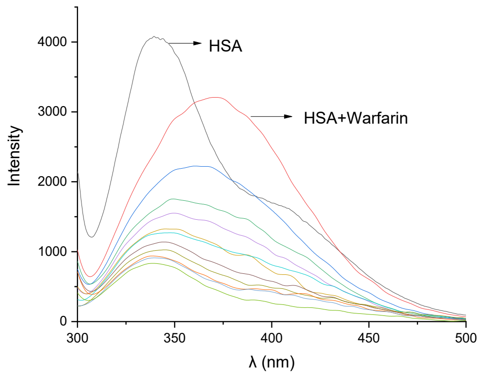

4′–NO2–fenH

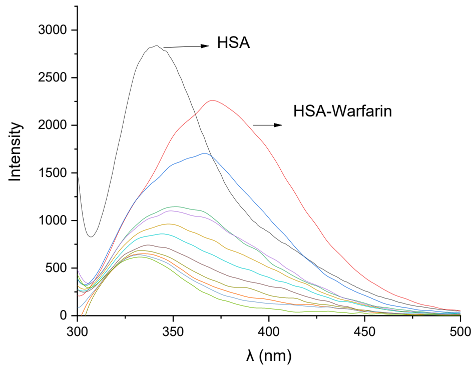

Complex **1**

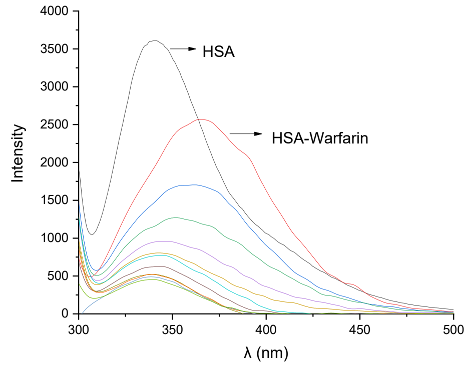

Complex **2**

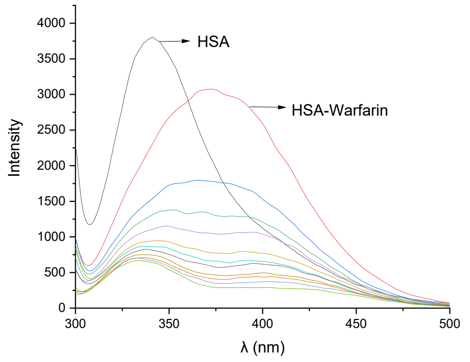

Complex **3**

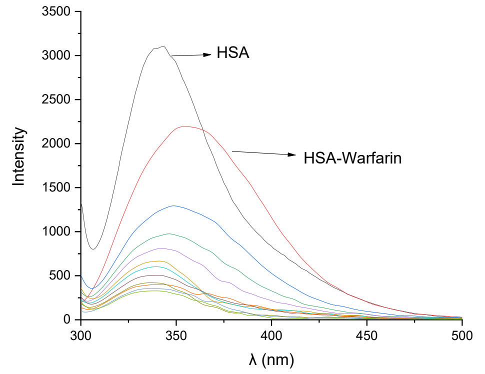

Complex **4**

Figure S23. Scatchard plots of the BSA quenching experiments in the presence of ibuprofen upon addition of the compounds.

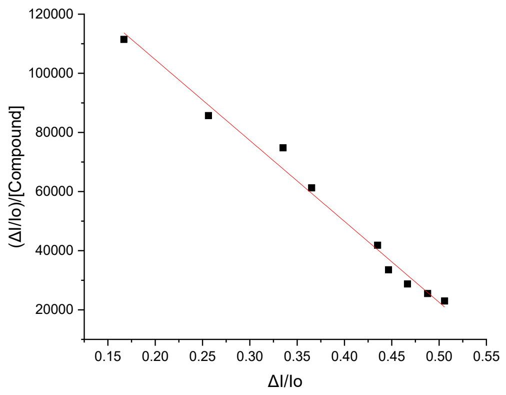

4′–NO_2–fenH

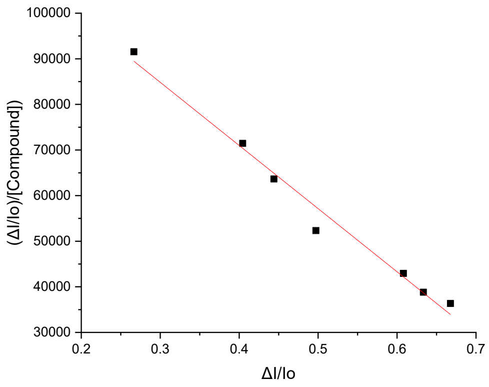

Complex **1**

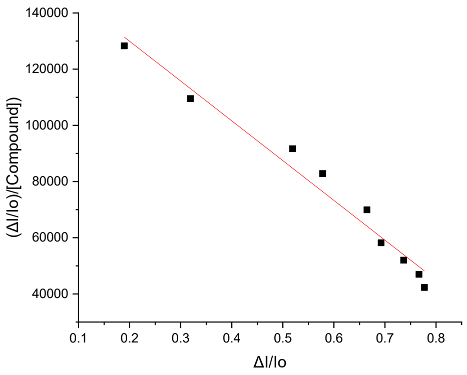

Complex **2**

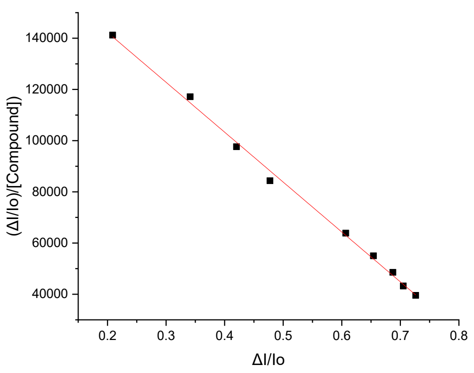

Complex **3**

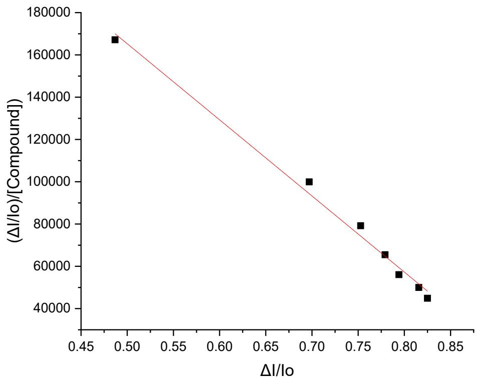

Complex **4**

Figure S24. Scatchard plots of the BSA quenching experiments in the presence of warfarin upon addition of the compounds.

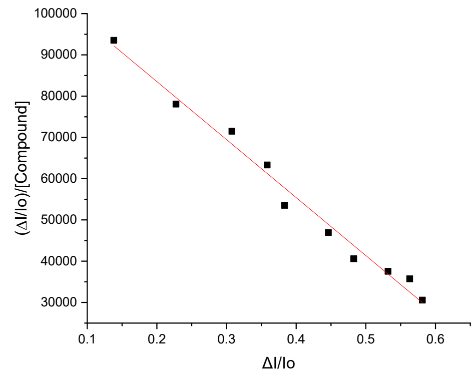

4′–NO_2–fenH

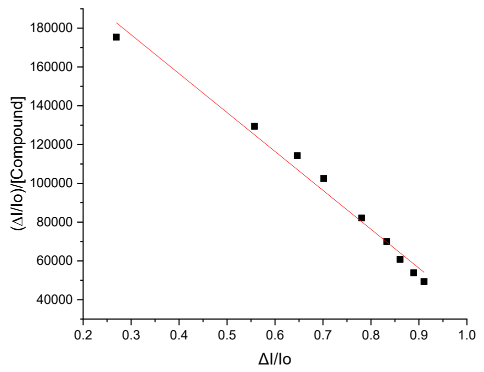

Complex **1**

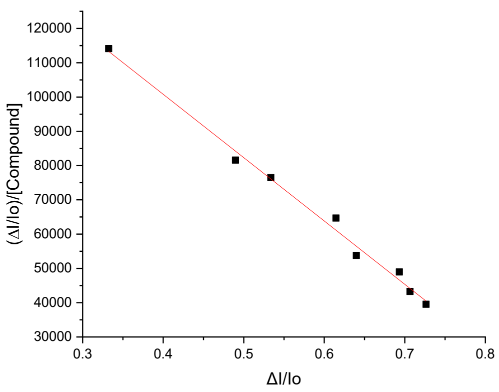

Complex **2**

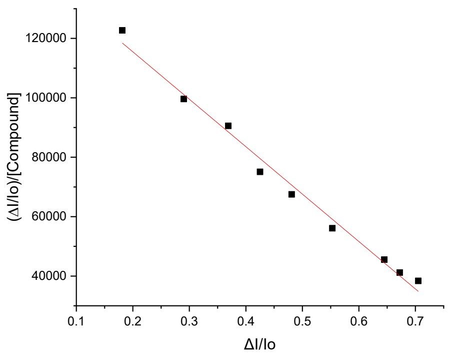

Complex **3**

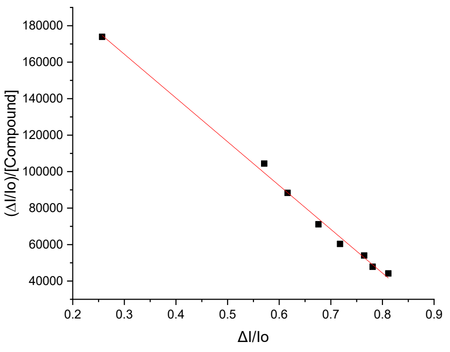

Complex **4**

Figure S25. Scatchard plots of the HSA quenching experiments in the presence of ibuprofen upon addition of the compounds.

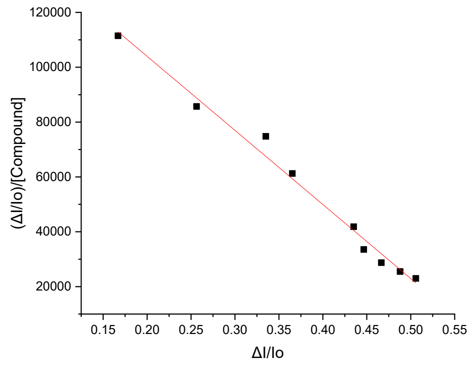

4′–NO_2–fenH

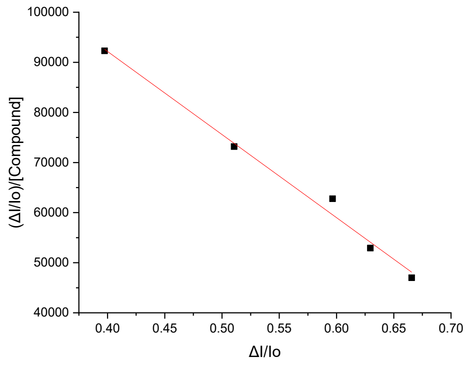

Complex **1**

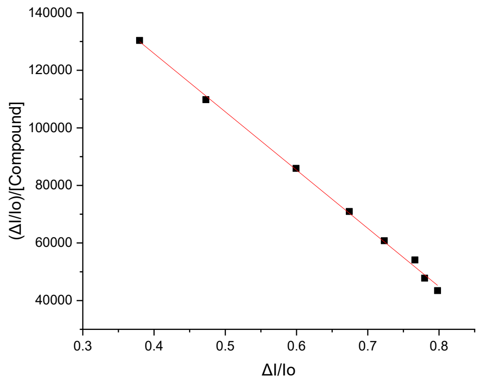

Complex **2**

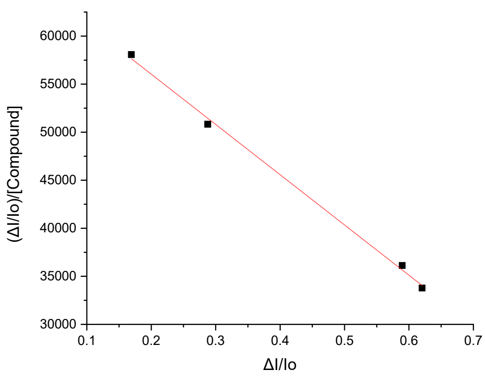

Complex **3**

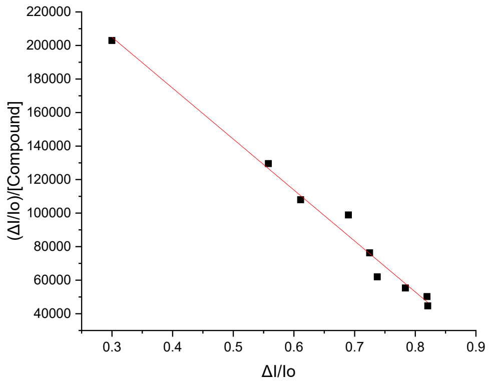

Complex **4**

Figure S26. Scatchard plots of the HSA quenching experiments in the presence of warfarin upon addition of the compounds.

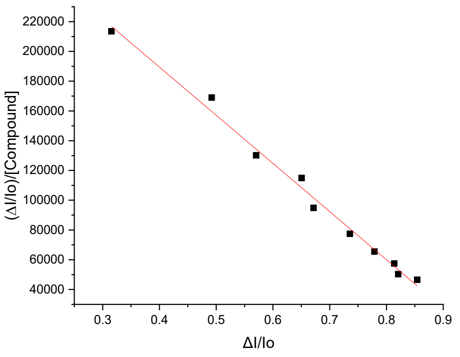

4′–NO_2–fenH

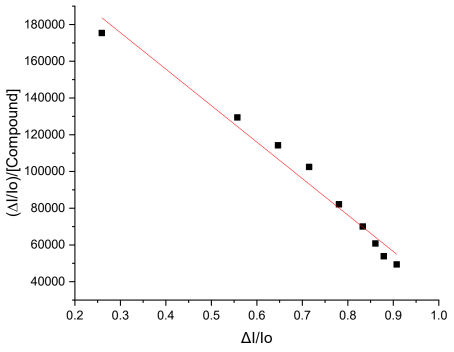

Complex **1**

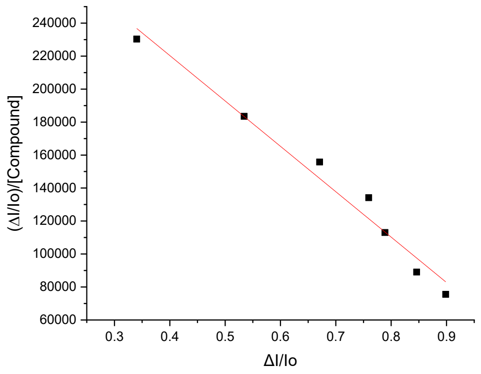

Complex **2**

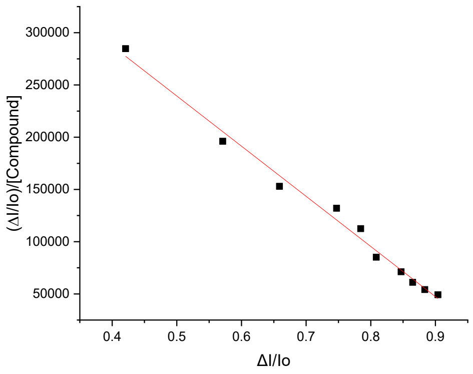

Complex **3**

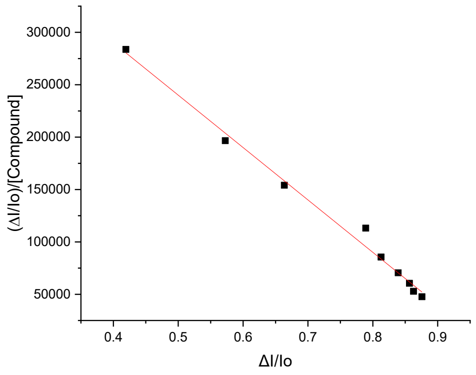

Complex **4**
